# Gene and Protein Expression in Response to Different Growth Temperatures and Oxygen Availability in *Burkholderia thailandensis*

**DOI:** 10.1371/journal.pone.0093009

**Published:** 2014-03-26

**Authors:** Clelia Peano, Fabrizio Chiaramonte, Sara Motta, Alessandro Pietrelli, Sebastien Jaillon, Elio Rossi, Clarissa Consolandi, Olivia L. Champion, Stephen L. Michell, Luca Freddi, Luigi Falciola, Fabrizio Basilico, Cecilia Garlanda, Pierluigi Mauri, Gianluca De Bellis, Paolo Landini

**Affiliations:** 1 Institute of Biomedical Technologies, National Research Council, Segrate, Milan, Italy; 2 Department of Biosciences, Università degli Studi di Milano, Milan, Italy; 3 Humanitas Clinical and Research Center Institute, Rozzano, Milan, Italy; 4 College of Life and Environmental Sciences, University of Exeter, Exeter, United Kingdom; 5 Department of Chemistry, Università degli Studi di Milano, Milan, Italy; Tulane University School of Medicine, United States of America

## Abstract

*Burkholderia thailandensis*, although normally avirulent for mammals, can infect macrophages *in vitro* and has occasionally been reported to cause pneumonia in humans. It is therefore used as a model organism for the human pathogen *B. pseudomallei*, to which it is closely related phylogenetically. We characterized the *B. thailandensis* clinical isolate CDC2721121 (BtCDC272) at the genome level and studied its response to environmental cues associated with human host colonization, namely, temperature and oxygen limitation. Effects of the different growth conditions on BtCDC272 were studied through whole genome transcription studies and analysis of proteins associated with the bacterial cell surface. We found that growth at 37°C, compared to 28°C, negatively affected cell motility and flagella production through a mechanism involving regulation of the flagellin-encoding *fliC* gene at the mRNA stability level. Growth in oxygen-limiting conditions, in contrast, stimulated various processes linked to virulence, such as lipopolysaccharide production and expression of genes encoding protein secretion systems. Consistent with these observations, BtCDC272 grown in oxygen limitation was more resistant to phagocytosis and strongly induced the production of inflammatory cytokines from murine macrophages. Our results suggest that, while temperature sensing is important for regulation of *B. thailandensis* cell motility, oxygen limitation has a deeper impact on its physiology and constitutes a crucial environmental signal for the production of virulence factors.

## Introduction

The *Burkholderia* genus includes several environmental species and one obligate pathogen, *B. mallei* [1]. Several *Burkholderia* species are “environmental pathogens”, *i.e.*, facultative pathogenic bacteria commonly found in the environment. For instance, the *Burkholderia cepacia* complex, an association of around 17 *Burkholderia* species, is able to infect several plants and can be a highly problematic opportunistic pathogen in cystic fibrosis patients [2]. Another very important environmental pathogen, *B. pseudomallei*, is commonly found in waters and wet soil in tropical regions. Upon entry into humans either through skin cuts or by inhalation, *B. pseudomallei* can cause melioidosis, a disease with multiple symptoms and a complex outcome, including acute pneumonia and septic shock, often fatal for the patient [3]. *B. pseudomallei* was initially thought to exist in two different biotypes, one able to cause melioidosis in humans, the second avirulent and distinguishable from the other by its ability to utilize arabinose as a carbon source [4]. This avirulent biotype was later classified as a separate species, *B. thailandensis* [5]. Although generally considered an environmental saprophyte, *B. thailandensis* has been reported to cause occasional pneumonia and melioidosis-like symptoms in humans, especially in victims of traumatic injuries [6], [7]. Comparison between *B. thailandensis* and *B. pseudomallei* genomes has highlighted several features that might be responsible for the strong difference in virulence between the two species, such as the presence, in *B. pseudomallei*, of a *Yersinia*-like fimbrial cluster and of a specific capsular polysaccharide [8]–[10]. However, it has recently been shown that some *B. thailandensis* strains, despite being able to produce a *B. pseudomallei*-like (Bp-like) capsule, are avirulent in mouse infection models, suggesting that additional factors are responsible for the difference in virulence between the two species [10], [11]. On the other hand, genomic studies have shown that, despite its poor pathogenicity in mammals, *B. thailandensis* shares a large set of putative virulence factors with *B. pseudomallei* [12], [13]. Indeed, *B. thailandensis* can induce formation of multi-nucleated giant cells (MNGC) in macrophage infection models *in vitro*; in addition, it is highly virulent in experimental infection models utilizing either nematodes or insects [11], [14]–[16].

Thus, *B. thailandensis* represents an intriguing example of a “transition state” between an environmental bacterium and a human pathogen, making it a suitable model for the study of *B. pseudomallei*. In this work, we studied the *B. thailandensis* strain CDC2721121, a clinical isolate identified as the etiologic agent of a pleural infection in a 76-year old male [6] and investigated its response to environmental signals through determination of global gene expression and cell surface-associated protein production. In particular, we studied the effects of oxygen availability and of different growth temperatures (28°C versus 37°C), *i.e.*, environmental conditions typical of the human host, but that can also be encountered by *B. thailandensis* in its natural niche (water and soil in tropical and subtropical regions). We found that changes in both temperature and oxygen availability trigger specific responses in global gene expression and in cell surface protein composition in *B. thailandensis*: in particular, growth at 37°C inhibits production of flagella and cell motility, while oxygen limitation promotes expression of factors involved in bacteria-host interactions.

## Results

### Genome Sequence of *B. thailandensis* CDC2721121 (BtCDC272)

**Figure 1:**
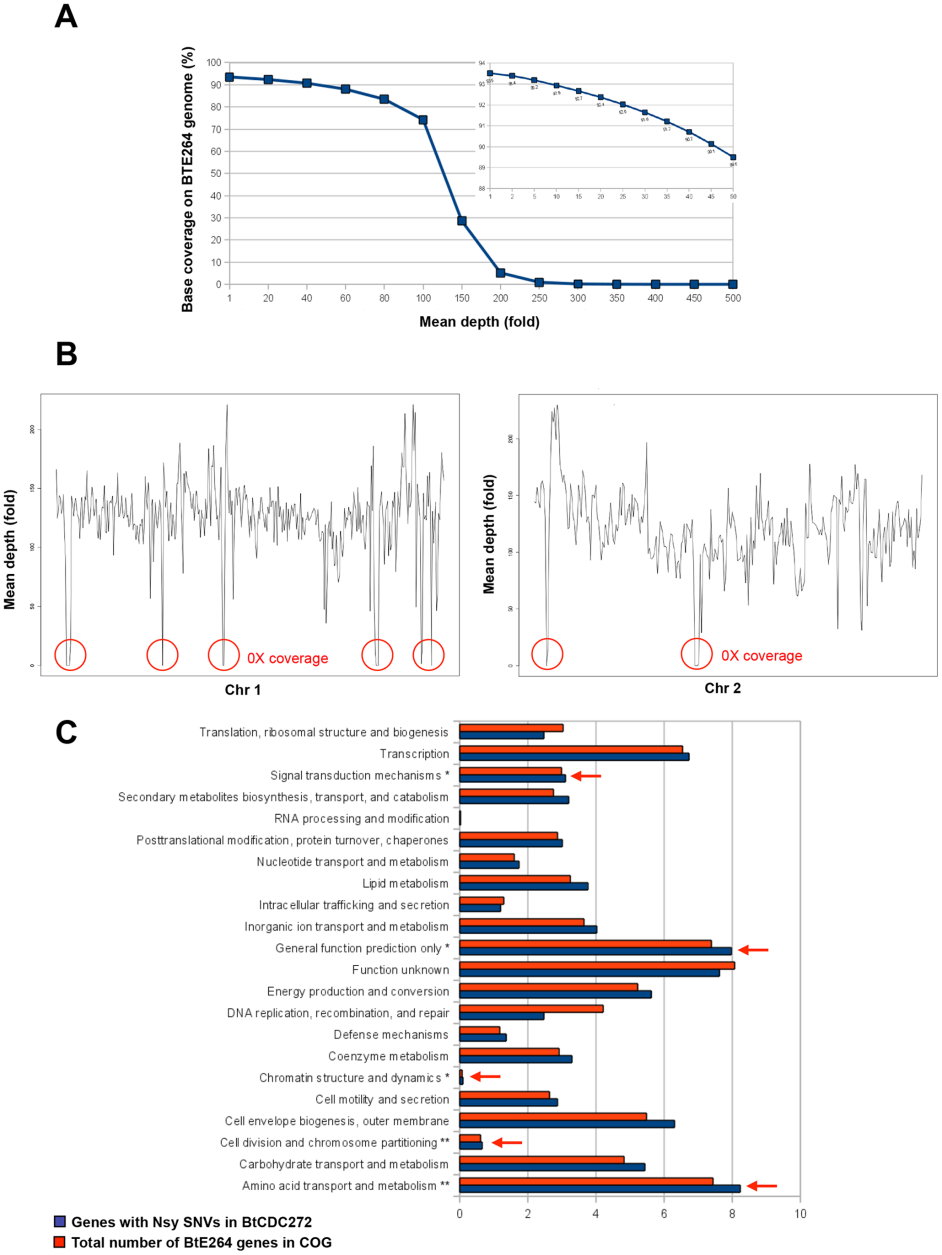
Genome sequencing of *B. thailandensis* BtCDC272. (**a**) Reference genome coverage. BtCDC272 reads were aligned against the BtE264 reference. Y-axis: percentage nucleotide coverage of the BtE264 genome. X-axis: number of reads covering each nucleotide. The inset graph indicates range from 1 to 50X coverage; ca. 90% of the BtE264 genome was sequenced with a 50X read depth. (**b**) Sequence coverage gaps in BtE264 are associated with GIs and nGis. Shown are coverage maps of BtE264 chromosome 1 (left) and chromosome 2 (right). Graph: median fold-coverage of BtCDC272 sequence reads using a 10-kb moving window. Red circles evidence the regions with a median 0X coverage within the entire 10 Kb moving window. The x-axis shows the BtE264 genome co-ordinates (bp). (**c**) Functional enrichment of Bt genes with nonsynonymous SNPs (Nsy SNPs). COG functional categories are indicated on the y-axis, and the percentage of genes in each COG category is shown on the x-axis. Dark blue columns represent BtCDC272 genes with Nsy SNPs relative to BtE264, and red columns indicate all BtE264 genes with COG annotations. COG categories exhibiting a significant enrichment of genes with Nsy SNPs are highlighted by asterisks (*P<0.05 or **P<0.01, binomial test; after Bonferroni correction).

*B. thailandensis* CDC2721121 (BtCDC272), unlike other *B. thailandensis* clinical isolates, does not produce the Bp-like capsule, but an exopolysaccharide more typical of strictly environmental *B. thailandensis* strains (Bt-EPS) [10], thus making this strain very interesting for the identification of additional virulence mechanisms. To gather more information about BtCDC272, we obtained its genomic sequence by performing Illumina paired-end deep sequencing. Through generation of more than 10 Million reads, we were able to map about 1 Gb of BtCDC272 genomic sequence to the corresponding sequences in the environmental isolate *B. thailandensis* E264 (BtE264), whose genome has been entirely sequenced and characterized [12] (GeneBank Accession number: CP000086, CP000085). Comparison to the BtE264 reference genome showed that 92.58% of Chromosome 1 was sequenced with a 124X-coverage and 94.63% of Chromosome 2 was sequenced with a depth of about 119X-coverage, thus obtaining a mean coverage of the whole BtE264 genome of 93.51% with a depth of coverage of about 122X (Figure 1A). Mapping the BtCDC272 Illumina sequencing reads to the BtE264 genome revealed the presence of 193 regions greater than 100 bp showing 0X coverage, for a total of 428.7 Kbp. Figure 1B shows the median coverage of BtE264 chromosomes 1 and 2 using a 10 Kb moving window; the regions with a median 0X coverage which span the entire moving window are highlighted by red circles. 78 of these 0X coverage regions (40.4%) were located inside previously identified genomic islands (GI) and novel genomic islets (nGI) regions [10], *i.e.*, chromosomal regions associated with horizontal gene transfer and exhibiting unusual sequence features, such as atypical codon bias or GC content, or the presence of multiple prophage and transposon-related genes (File S1; sheet “0X coverage regions in Gi-nGi”). Such regions are often composed of mobile DNA elements, leading to their frequent loss or acquisition in a strain-specific manner. 87 of the remaining 0X coverage regions overlap with 89 BtE264 CDS for at least 50% of their length, suggesting that these genes might be absent in BtCDC272 (File S1; sheet “novel 0X coverage regions”). Interestingly, 43 of these 89 CDS encode for putative transposases and, although not part of any GI region, are also related to DNA mobile elements. The remaining sequences, encompassing 46 genes belonging to different functional categories, were often adjacent to genes encoding transposases. Interestingly, among BtE264 genes missing in BtCDC272 we found a gluconate transport system (BTH_II0352-0353, flanking a putative transposase-encoding gene, BTH_II0354) and two gene clusters encoding putative rhamnosyltransferases possibly involved in rhamnolipid biosynthesis: BTH_II1075-1081, flanking the transposase-encoding BTH_II1082 gene, and BTH_II1875-1880. It is noteworthy that rhamnolipid production is related to virulence in several opportunistic pathogens such as *Pseudomonas aeruginosa* [17] and that different ability in sugar (arabinose) utilization is a distinctive feature of *B. thailandensis* vs. *B. pseudomallei* [4]. Finally, 28 0X coverage regions are BtCDC272 specific sequences not matching to the BtE264 genome (File S1; sheet “novel 0X coverage regions”).

Altogether, *de novo* assembly of the BtCDC272 reads yielded 594 sequence contigs with median size (N50) of 41229 bases. The annotation of the 594 contigs generated 6141 CDS, 5212 of which, showing more than 30% of protein sequence identity over more than 80% of the protein length, were considered as matching genes present in the BtE264 genome. In contrast, 929 CDS appeared to be specific for BtCDC272 (File S2). Similar to other *Burkholderia* genomes, we found frequent duplications of genes and operons (often found in one copy for each chromosome), such as for genes encoding flagellum biosynthetic proteins, ATP synthase, aspartate carbamoyltransferase, etc. A homology search for the 929 CDS specific for BtCDC272 was performed using a *Burkholderia* database containing 98044 protein sequences from 4 *B. mallei*, 9 *B. pseudomallei* and 3 *B. thailandensis* strains; 331 BtCDC272-specific CDS showed very high similarity (match >80% protein length - Identity >30%) with CDS from other *Burkholderia* species, mainly with *B. thailandensis* CDC3015869 (File S3), thus reiterating the close phylogenetic relation among *B. thailandensis* clinical isolates [10]. Of the 929 BtCDC272-specific CDS only 245 could tentatively be assigned to functional categories, the most represented being: Carbohydrate transport and metabolism (15 CDS), Transcription (24 CDS), Replication, recombination and repair (33 CDS), Cell wall/membrane/envelope biogenesis (17 CDS), and Secondary metabolites biosynthesis, transport and catabolism (14 CDS). Among the genes belonging to the “Replication, recombination and repair” category, the majority coded for integrases and transposases, once again underlining the correlation between strain-specific genes and mobile DNA elements. Finally, 19 genes, found on two different contigs, encode phage assembly proteins, suggesting the presence of at least one prophage on the BtCDC272 genome. Interestingly, similar phage-like gene clusters were found on the genome of the *B. thailandensis* CDC3015869 strain, suggesting that these two clinical isolates of *B. thailandensis* might carry the same prophage. In addition to genes homologous to other *Burkholderia* species, several genes possibly involved in Cell wall/membrane/envelope biogenesis were highly similar to other Gram-negative bacteria, like *Salmonella typhimurium* and *Caulobacter vibrioides,* while genes involved in Secondary metabolites biosynthesis showed significant similarity to various *Bacillus subtilis* polyketide synthases. Thus, genome sequencing of BtCDC272 confirmed once again the elevated plasticity of the *Burkholderia* genomes.

Comparing regions conserved between BtE264 and BtCDC272 genomes, we identified 44,613 Single Nucleotide Variations (SNVs), approximately 77.5% of which (34595 SNVs) occurred within coding sequences. Non-synonymous SNVs amounted to 17,121, yielding an overall intragenic nucleotide diversity between BtE264 and BtCDC272 of approximately 5.9 SNV per kilobase. When mapped across various functional pathways (COG categories), the percentage of genes containing non-synonymous SNVs (SNVs predicted to alter protein sequence) were found to be significantly higher (q-value<0.05, Fisher Exact Test, after multiple hypothesis Bonferroni correction) in pathways related to signal transduction mechanisms, chromatin structure and dynamics, cell division, and amino acid transport and metabolism (Figure 1C). Interestingly, two BtCDC272 genes belonging to the Bt-EPS biosynthetic cluster, and homologous to the BtE264 genes BTH_I1343 and BTH_I1339, show a significantly higher mutation frequency (12.87 and 7.66 SNV/kbp, respectively) than the average. It remains to be understood whether such a high mutation frequency might result in altered enzymatic catalytic activity or substrate affinity, thus affecting composition and structure of the Bt-EPS in BtCDC272 (File S4).

### Gene and Protein Expression Changes in BtCDC272 in Response to Different Temperatures and to Oxygen Limitation

In order to investigate the response of *B. thailandensis* to environmental signals, we compared BtCDC272 global gene expression in different growth conditions, namely, in LB growth medium with full aeration at either 28°C or 37°C, and at 37°C in oxygen limitation, *i.e.*, in liquid cultures incubated with no shaking at 37°C (abbreviated 37°C An, for “anoxic”, in figures and tables). In cultures grown at 37°C with no shaking, concentration of dissolved oxygen in liquid medium decreased rapidly from the initial value of around 5 mg/l, falling below detection limits in stationary phase cells (Figure S1B), as determined by direct oxygen measurement using a galvanic sensor for dissolved oxygen in liquids (see *Methods*). Lack of oxygen results in earlier growth arrest and in production of lower biomass (Figure S1A); indeed, LB medium is not able to support anaerobic respiration by BtCDC272, unless supplemented with additional nitrate (data not shown). In contrast, oxygen concentration remained constant at all times in cultures grown with vigorous shaking (Figure S1B). Fluctuations in temperature and oxygen availability similar to those analyzed in our study are typical of *B. thailandensis* environment, *i.e.*, in soil and water bodies of tropical and subtropical regions. For instance, in pond waters in Thailand, the average temperature is estimated at 31.6°C [18] and oxygen concentrations can drop resulting in microaerophilic or even anoxic conditions. Upon entry to a human host, environmental bacteria experience a shift to 37°C, initially in an aerobic niche (*e.g.*, skin wounds, or nostrils upon inhalation) and then, progressing in the host tissues or bloodstream, into a microaerophilic/anoxic environment. Thus, the growth conditions chosen in this work aimed to compare BtCDC272 gene expression in conditions resembling human host colonization versus conditions more typical of the external environment. Samples for RNA sequencing (RNA-seq) were taken from stationary phase BtCDC272 cultures (grown 16 hours starting from an inoculum of OD600 nm = 0.1; Figure S1); additional details are provided in *Methods*. The statistics regarding RNA-seq data generation are included in File S5. We chose to use bacteria in stationary phase, rather than actively growing cells, since their physiology is more similar to environmental conditions, typically oligotrophic and unable to support fast growth, and also to the situation encountered by bacteria in the host, in particular upon ingestion by macrophages [12]. Genes showing significantly differential expression (DEGs) (p-val≤0.01 and log2 fold change ≥1 or log2 fold change ≤−1) are listed in File S6 (37°C versus 28°C; 416 genes) and File S7 (37°C An versus 37°C; 618 genes). Analysis of functional classes affected by the different growth conditions showed that a significantly higher number of DEGs genes belonging to two functional classes, *i.e.*, “Cell motility and secretion” and “Signal transduction mechanisms” were less expressed at 37°C than at 28°C (Figure S2A): most genes belonging to the second category are in fact signal transduction proteins involved in chemotaxis (see Table 1), and thus also related to cell motility. In contrast, genes involved in primary metabolism (Functional classes “Energy production and conversion”, “Nucleotide transport and metabolism” and “Translation, ribosomal structure and biogenesis”) were significantly more expressed in anoxic than in fully aerated conditions (Figure S2B). This result was unexpected, since expression of primary metabolism genes, in particular of those encoding ribosomal proteins, usually correlates with growth rate (reviewed in [19]) and is therefore higher in optimal growth conditions. However, in our experiments, we used stationary phase cells (Figure S1A), in which growth-rate regulation should not come into play. A more detailed analysis of the effects of oxygen limitation on ribosomal gene expression is provided in “*Validation of functional genomics experiments by Real-Time PCR*” (see below). Differentially Expressed Genes (DEGs) and Outer membrane Proteins (DEPs) most relevant to specific cellular processes are listed in Table 1 (37°C versus 28°C) and Table 2 (37°C An versus 37°C) and are discussed in the following subsections.

**Table 1:** Gene and protein regulation in response to different growth temperatures (28°C vs. 37°C).

Gene ID(name)	Gene product	RNAseq		MudPIT	Conservation^d^	Organization in operons and previously observed regulation^e^
GENES/PROTEINS DOWNREGULATED AT 37°C		FC^a^	log_2_(FC)^b^	DAve^c^		
**Motility (12)**						
BTH_I0200 (*fliF*)	flagellar MS-ring protein			−100	All	
BTH_I0202 (*fliS*)	flagellin specific chaperone	0.35	−1.5		All	[21]
BTH_I0237	flagellar biosynthesis chaperone	0.13	−2.9		All	BTH_I0237→*flgM* [21]
BTH_I0238 (*flgM*)	Anti σ^28^ factor; regulator offlagellin synthesis	0.31	−1.7		All	BTH_I0237→*flgM* [21]
BTH_I0243 (*flgE*)	flagellar hook protein			−94.2	All	
BTH_I0250 (*flgK*)	flagellar hook associated protein	0.31	−1.7		All	*flgK*→*flgL* [21]
BTH_I0251 (*flgL*)	flagelin and related proteins	0.38	−1.4		All	*flgK*→*flgL* [21]
BTH_I3184 (*motB*)	flagellar motor protein	0.19	−2.4		All	*motA*→*motB* [21]
BTH_I3185 (*motA*)	flagellar motor component	0.11	−3.2		All	*motA*→*motB* [21]
BTH_I3196 (*fliC*)	flagellin	0.38	−1.4	−110.2	All	[21]
BTH_I3197 (*fliD-1*)	flagellar capping protein	0.33	−1.6		All	[21]
BTH_II1544 (*fliD-2*)	flagellar capping protein	0.50	−1		Bp, Bm	[21]
**Chemotaxis (12)**						
BTH_I3175 (*cheZ*)	chemotaxis protein histidine kinase	0.25	−2		All	*cheZ*→*cheA* [21]
BTH_I3176 (*cheY*)	chemotaxis regulatory protein	0.50	−1		All	*cheZ*→*cheA* [21]
BTH_I3177 (*cheB*)	chemotaxis response regulator	0.25	−2		All	*cheZ*→*cheA* [21]
BTH_I3178 (*cheD*)	chemoreceptor protein	0.14	−2.8		All	*cheZ*→*cheA* [21]
BTH_I3179 (*cheR*)	chemotaxis methyltransferase	0.08	−3.6		All	*cheZ*→*cheA* [21]
BTH_I3180	methyl-accepting chemotaxis protein	0.20	−2.3		All	*cheZ*→*cheA* [21]
BTH_I3181 (*cheW*)	chemotaxis signal transduction protein	0.11	−3.2		All	*cheZ*→*cheA* [21]
BTH_I3182 (*cheA*)	chemotaxis protein histidine kinase	0.27	−1.9		All	*cheZ*→*cheA* [21]
BTH_I3209	tar, methyl-accepting chemotaxis protein	0.47	−1.1		All	[21]
BTH_II1464	tar, methyl-accepting chemotaxis protein	0.31	−1.7		All	[21]
BTH_II1955	tar, methyl-accepting chemotaxis protein	0.38	−1.4		Bp	
BTH_II2364 (*cheC*)	chemotaxis protein, MCP inhibitor	0.33	−1.6		Bp, Bc	[21]
**ATP synthesis (6)**						
BTH_II0419 (*atpD-2*)	ATP synthase F_1_-complex, beta subunit	0.29	−1.8		Bp, Bm	*atpD*-2→*atpG-2*
BTH_II0420 (*atpC-2*)	ATP synthase F_1_-complex, epsilon subunit	0.33	−1.6		Bp, Bm	*atpD*-2→*atpG-2*
BTH_II0423 (*atpB-2*)	ATP synthase F_0_ complex, a subunit	0.35	−1.5		Bp, Bm	*atpD*-2→*atpG-2*
BTH_II0424 (*atpE-2*)	ATP synthase F_0_ complex, c subunit	0.38	−1.4		Bp, Bm	*atpD*-2→*atpG-2*
BTH_II0425 (*atpF-2*)	ATP synthase F_0_ complex, b subunit	0.44	−1.2		Bp, Bm	*atpD*-2→*atpG-2*
BTH_II0427 (*atpG-2*)	ATP synthase F_1_ complex gamma subunit	0.47	−1.1		Bp, Bm	*atpD*-2→*atpG-2*
**Nitrogen metabolism (6)**						
BTH_I1851 (*narI*)	nitrate reductase, gamma subunit	0.38	−1.4		Bp, Bm	*narI*→BTH_I1856 [21]
BTH_I1852 (*narJ*)	nitrate reductase, delta subunit	0.41	−1.3		Bp, Bm	*narI*→BTH_I1856 [21]
BTH_I1855 (*narK*)	nitrate transporter	0.44	−1.2		Bp, Bm	*narI*→BTH_I1856 [21]
BTH_I2321 (*nosL*)	lipoprotein involved in nitrous oxide reduction	0.47	−1.1		Bp	BTH_I2317→*nosD*
BTH_I2324 (*nosD*)	periplasmic copper binding protein	0.50	−1		Bp	BTH_I2317→*nosD*
BTH_I2325 (*nosZ*)	nitrous oxide reductase activity	0.33	−1.6		Bp	
**Universal stress proteins (USP) (6)**						
BTH_II1566	universal stress protein UspA	0.25	−2		All	
BTH_II1567	universal stress protein UspE	0.31	−1.7		All	BTH_II1567→II1568
BTH_II1568	universal stress protein UspA	0.35	−1.5		All	BTH_II1567→II1568
BTH_II1569	universal stress protein UspA	0.47	−1.1		All	
BTH_II1268	universal stress protein UspA	0.31	−1.7		Bp,Bc	
BTH_II0442	universal stress protein UspA	0.50	−1		Bp,Bc	
**GENES/PROTEINS UPREGULATED AT 37°C**						
**Chaperones (3)**						
BTH_I1308 (*dnaK*)	molecular chaperone DnaK	2	1		All	[21]
BTH_I1309 (*dnaJ*)	molecular caperone DnaJ	2.30	1.2		All	[21]
BTH_I1458 (*groL*)	chaperonin GroEL			99	All	
**Protein secretion systems/exoenzymes (6)**						
BTH_I0028 (*fliN*)	flagellar motor switch/type III secretory pathway protein	2.30	1.2		All	[21]
BTH_I0198 (*fliH*)	flagellar biosynthesis protein/type III secretory pathway protein	2.14	1.1		All	[21]
BTH_II0191 (*flgI*)	flagellar basal-body P-ring protein	2.30	1.2		Bp, Bc	
BTH_I2671 (*plcC*)	acid phosphatase	2	1		All	
BTH_I2962 (*hcp*)	hemolysin coregulated protein, T6SS component			67	Bp,Bc	
BTH_I2963	uncharacterized T6SS protein			100	Bc, Bp	

a FC = fold change in differential expression;

b log_2_(FC) = logarithm_2_ of fold change.

c DAve = Difference in Average (amount); calculated as in Experimental procedures.

d Conservation in *Burkholderia* species pathogenic for humans: Bc = *B. cenocepacia*; Bm = *B. mallei*; Bp = *B. pseudomallei*.

e in the reference indicated, the gene or its orthologous has been found with the same trend of expression in growth conditions comparable with this work. Genes and corresponding proteins found to be differentially expressed both in transcriptomics and proteomics experiments are underlined.

**Table 2:** Gene and protein regulation in response to oxygen-limiting conditions.

Gene ID (name)	Gene product	RNAseq		MudPIT	Conservation^d^	Organization in operons and previously observed regulation^e^
GENES/PROTEINS DOWNREGULATED IN OXYGEN LIMITATION		FC^a^	log_2_(FC)^b^	DAve^c^		
**Motility (1)**						
BTH_I3196 (*fliC*)	Flagellin			−98.5	All	[33]
**Extracellular proteins (6)**						
BTH_I0090 (*cpxP*)	P pilus assembly/Cpx signaling pathway, periplasmic inhibitor	0.41	−1.3	−150	Bp, Bm	
BTH_I0866 (*fimC*)	P pilus assembly protein, chaperone PapD	0.29	−1.8		All	
BTH_I2461 (*cpaA*)	Flp pilus assembly protein, protease CpaA	0.47	−1.1		Bp, Bm	
BTH_II1990	putative outer membrane	0.38	−1.4	−150	Bp	
BTH_II0447	TolC-like outer membrane protein/RND efflux system component	0.50	−1		Bp, Bm	
BTH_II1638	H-type lectin domain containing protein			−184.6	Bp, Bm	
**Polyhydroxyalkanoates synthesis (3)**						
BTH_I2255 (*phaC*)	poly(3-hydroxyalkanoate) synthetase			−37.4	All	
BTH_I2258 (*phaR*)	polyhydroxyalkanoate synthesis repressor			−120.5	Bp, Bc	
BTH_II0418 (*phaC*)	poly(3-hydroxyalkanoate) synthetase			−113.9	All	
**Universal stress proteins (USP) (6)**						
BTH_II1566	universal stress protein UspA	0.44	−1.2		All	
BTH_II1567	universal stress protein UspE	0.35	−1.5		All	BTH_II1567→II1568
BTH_II1568	universal stress protein UspA	0.23	−2.1		All	BTH_II1567→II1568
BTH_II1569	universal stress protein UspA	0.44	−1.2		All	
BTH_II1268	universal stress protein UspA	0.31	−1.7		Bp,Bc	
BTH_II2484	universal stress protein UspA	0.22	−2.2		All	
**Sulfur assimilation (5)**						
BTH_I0817 (*cysD*)	sulfate adenylyltransferase	0.29	−1.8		All	[33]
BTH_I2478 (*cysW*)	ABC-type sulfate transport system, permease component	0.50	−1		All	[33]
BTH_I2500 (*ssuC/tauC*)	alkanesulfonate transporter permease subunit	0.50	−1		All	
BTH_II1370	cystathionine beta-synthase	0.31	−1.7		None	
BTH_II2316 (*sulP*)	sulfate permease	0.44	−1.2		All	
**GENES/PROTEINS UPREGULATED IN OXYGEN LIMITATION**						
**Chaperones (7)**						
BTH_I0955 (*htpG*)	chaperone HSP90 family			116.5	All	[33]
BTH_I0977 (*hslJ*)	HslJ, heat shock protein	2.14	1.1		All	
BTH_I1306 (*grpE*)	molecular chaperone GrpE	2.64	1.4		All	[33]
BTH_I1308 (*dnaK*)	molecular chaperone DnaK			193.2	All	[33]
BTH_I1457 (*groS*)	co-chaperonin GroES	4.59	2.2	23.5	All	*groS*→*groL* [33]
BTH_I1458 (*groL*)	chaperonin GroEL	2.46	1.3	99	All	*groS*→*groL* [33]
BTH_I1878 (*hscB*)	co-chaperone HscB	2.00	1		All	[33]
**Ribosomal proteins and RNA polymerase (54)**						
BTH_I0473 (*rplY*)	ribosomal protein L25	6.50	2.7	150	All	
BTH_I0735 (*rpsT*)	S20	4.59	2.2		All	[33]
BTH_I0779 (*rpmG*)	L33	3.73	1.9		All	*rpmG*→*rpmB*
BTH_I0780 (*rpmB*)	L28	3.73	1.9	150	All	*rpmG*→*rpmB*
BTH_I1055 (*rpsO*)	S15P/S13E	2.30	1.2		All	
BTH_I1140 (*rplU*)	L21	4.29	2.1		All	*rplU*→*rpmA*
BTH_I1141 (*rpmA*)	L27	6.50	2.7		All	*rplU*→*rpmA*
BTH_I1233 (*rplM*)	L13	4.29	2.1		All	*rplM*→*rpsI*
BTH_I1234 (*rpsI*)	S9	3.03	1.6	26.5	All	*rplM*→*rpsI*
BTH_I1638 (*rpsA*)	S1	4.29	2.1	142.8	All	
BTH_I1661 (*rpsP*)	S16	2.83	1.5	83.5	All	
BTH_I1664 (*rplS*)	L19	5.28	2.4	133.8	All	
BTH_I1715 (*rpmF*)	L32	3.73	1.9		All	
BTH_I2027 (*rpsB*)	S2	4.00	2	132.5	All	[33]
BTH_I2179 (*rpsF*)	S6	6.96	2.8		All	*rpsR*→*rplI* [33]
BTH_I2181 (*rpsR*)	S18	3.48	1.8		All	*rpsR*→*rplI* [33]
BTH_I2182 (*rplI*)	L9	4.92	2.3		All	
BTH_I2213 (*rpmE*)	L31	4.92	2.3	150	All	
BTH_I2592 (*rplT*)	L20	4.59	2.2		All	*rplT*→*rpmI* [33]
BTH_I2593 (*rpmI*)	L35	3.48	1.8		All	*rplT*→*rpmI* [33]
BTH_I3041 (*rplQ*)	L17	3.25	1.7		All	
BTH_I3043 (*rpsD*)	S4	3.73	1.9	129.1	All	[33]
BTH_I3049→BTH_I3058 (*rplO* →*rplN*;10 genes)	L15, L30, S5,L18, L6, S8, S14, L5, L24, L14 (6 proteins)	4→15	2→3.9	83→141	All	*rplO*→*rplN* [33]
BTH_I3059→BTH_I3068 (*rpsQ* →*rpsC*;10 genes)	S17,L29, L16, S3, L22, S19, L2, L23, L4, L3 (5 proteins)	3→14	1.6→4	48→139	All	*rpsQ*→*rplC*
BTH_I3069 (*rpsJ*)	S10	9.85	3.3	61.4	All	
BTH_I3072 (*rpsG*)	S7	4.00	2		All	
BTH_I3073 (*rpsL*)	S12	4.29	2.1		All	
BTH_I3077 (*rplL*)	L7/L12	3.25	1.7	153.1	All	
BTH_I3078 (*rpllJ*)	L10	7.46	2.9	100	All	
BTH_I3079 (*rplA*)	L1	9.19	3.2		All	*rplA*→*rplK*
BTH_I3080 (*rplK*)	L11	7.46	2.9		All	*rplA*→*rplK*
BTH_I3195 (*rpsU-1*)	S21	3.73	1.9		All	[33]
BTH_II0618 (*rpsU-2*)	S21	3.73	1.9		All	[26], [33]
BTH_I3042 (*rpoA*)	DNA directed RNA polymerase alpha subunit	5.66	2.5		All	
BTH_I3075 (*rpoC*)	DNA directed RNA polymerase beta’ subunit	4.59	2.2		All	
BTH_I3077 (*rpoB*)	DNA directed RNA polymerase beta subunit	3.48	1.8		All	
**Motility (5)**						
BTH_I0200 (*fliF*)	flagellar biosynthesis lipoprotein			150	All	
BTH_I0241 (*flgC*)	flagellar basal body rod protein	2.30	1.2		All	[25]
BTH_I0242 (*flgD*)	flagellar hook capping protein	2.14	1.1		All	*flgD*→*flgE* [25], [33]
BTH_I0243 (*flgE*)	flagellar hook protein			33.4	All	*flgD*→*flgE* [25], [33]
BTH_II1544 (*fliD*)	flagellar capping protein			33.1	All	
**ATP-synthesis (8)**						
BTH_I3307 (*atpC-1*)	ATP synthase F_1_-complex, epsilon subunit	3.73	1.9		All	
BTH_I3308 (*atpD-1*)	ATP synthase F_1_-complex, beta subunit	4.92	2.3	110.2	All	*atpD-1*→*atpA-1*
BTH_I3309 (*atpG-1*)	ATP synthase F_1_-complex, gamma subunit	4.00	2		All	*atpD-1*→*atpA-1*
BTH_I3310 (*atpA-1*)	ATP synthase F_1_-complex, alpha subunit	4.00	2	100	All	*atpD-1*→*atpA-1*
BTH_I3311 (*atpH*)	ATP synthase F_1_-complex, delta subunit	5.28	2.4		All	*atpH*→*atpF*
BTH_I3312 (*atpF*)	ATP synthase F_0_ complex, b subunit	2.64	1.4		All	*atpH*→*atpF*
BTH_I3313	ATP synthase F_0_ complex, c subunit	4.92	2.3		All	
BTH_I3314 (*atpB-1*)	ATP synthase F_0_ complex, a subunit	2.14	1.1		All	
**Protein secretion systems/secreted proteins (11)**						
BTH_I2959 (*tssG-1*)	uncharacterized T6SS protein	2.30	1.2		All	*clpV-1*→*hcp-1*
BTH_I2960 (*tssF-1*)	uncharacterized T6SS protein	2.64	1.4		All	*clpV-1*→*hcp-1*
BTH_I2962 (*hcp-1*)	hemolysin coregulated protein	2.83	1.5	19.8	Bc, Bp	*clpV-1*→*hcp-1*
BTH_I2963 (*tssC-1*)	uncharacterized T6SS protein	2.64	1.4	163.6	Bc, Bp	*tssC-1*→*tagM-1* [26], [33]
BTH_I2964 (*tssB-1*)	hypothetical T6SS protein	2.83	1.5		Bc, Bp	*tssC-1*→*tagM-1* [26], [33]
BTH_I2965 (*tagM-1*)	uncharacterized T6SS protein	2.00	1		Bc, Bp	*tssC-1*→*tagM-1* [26], [33]
BTH_I2967 (*tssK-1*)	uncharacterized T6SS protein	2.83	1.5		Bc, Bp	
BTH_I3204	Lipoprotein	2.64	1.4		All	BTH_I3204→*csgG* [33]
BTH_I3205	lipoprotein	2.83	1.5		All	BTH_I3204→*csgG* [33]
BTH_I3206 (*csgG*)	lipoprotein, export of curli fibers	3.03	1.6	43.9	All	BTH_I3204→*csgG* [33]
BTH_II0816	zinc metalloprotease (elastase)	2.30	1.2		Bc, Bp	[26]
**Nucleotide biosynthesis (19)**						
BTH_I0474 (*prsA*)	phosphoribosylpyrophosphate synthetase	3.48	1.8	150	All	
BTH_I0739 (*adk*)	adenylate kinase	2.14	1.1		All	
BTH_I0860 (*dcd*)	deoxycytidine deaminase	2.30	1.2		All	
BTH_I1014 (*purD*)	phosphoribosylamine-glycine ligase	3.03	1.6		All	
BTH_I1056 (*pnp*)	polyribonucleotide nucleotidyltransferase	2.14	1.1	79.8	All	
BTH_I1153 (*nrdA*)	ribonucleotide reductase, alpha subunit	2.30	1.2	200	All	[33]
BTH_I1250 (*purH*)	AICAR transformylase/IMP cyclohydrolase	2.30	1.2		All	
BTH_I1317 (*purM*)	phosphoribosylaminoimidazole (AIR) synthetase	2.30	1.2		All	
BTH_I1465 (*pyrB*)	aspartate carbamoyltransferase	2.14	1.1		All	[33]
BTH_I1612 (*add*)	adenosine deaminase	2.14	1.1		All	[33]
BTH_I1892 (*pyrG*)	CTP synthase	2.14	1.1	150	All	
BTH_I2029 (*pyrH*)	uridylate kinase	2.14	1.1		All	
BTH_I2135 (*purL*)	phosphoribosylformylglycinamidine synthase			200	All	
BTH_I2231 (*ndk*)	nucleoside diphosphate kinase	3.25	1.7		All	[33]
BTH_I2245 (*purA*)	adenylosuccinate synthase	2.30	1.2		All	[33]
BTH_I2414 (*purN*)	phosphoribosylglycinamide formyltransferase	2.30	1.2		All	
BTH_I2511 (*pyrD*)	dihydroorotate dehydrogenase	2.83	1.5		All	
BTH_I3111 (*pyrE*)	orotate phosphoribosyltransferase	2.64	1.4		All	
BTH_II0686 (*purF*)	glutamine phospho ribosylpyrophosphate amidotransferase	2.64	1.4		All	[33]
**EPS/LPS biosynthesis (16)**						
BTH_I1125 (*lpxC*)	UDP-3-O-acyl-N-acetylglucosamine deacetylase	2.30	1.2		All	
BTH_I1470 (*rfbA*)	dTDP-glucose pyrophosphorylase			150	All	BTH_I1469→I1479
BTH_I1476 (*galE-1*)	UDP-glucose-4-epimerase	2	1		Bp, Bm	BTH_I1469→I1479
BTH_I1477	glycosyltransferase	2.14	1.1		All	BTH_I1469→I1479
BTH_I1478 (*wcaA*)	glycosyltransferases	2.64	1.4		Bm, Bp	BTH_I1469→I1479
BTH_I1485 (*galE-2*)	UDP-glucose-4-epimerase	2.14	1.1		All	
BTH_I1643 (*rfaE*)	ADP-heptose synthase, bifunctional sugar kinase/adenylyltransferase	2.30	1.2		All	
BTH_I1757 (*rfaG*)	glycosyltransferase	2	1		All	[33]
BTH_I1982 (*ydhO*)	cell wall hydrolase	2.30	1.2		All	[33]
BTH_I2035	outer membrane protective antigen OMA87	2.64	1.4		All	BTH_I2035→*lpxD*
BTH_I2036 (*hlpA*)	outer membrane protein	2.64	1.4		All	BTH_I2035→*lpxD*
BTH_I2037 (*lpxD*)	UDP-3-O-[3-hydroxymyristoyl] glucosamine N-acyltransferase	2.83	1.5		All	BTH_I2035→*lpxD*
BTH_I2038 (*fabA*)	3-hydroxymyristoyl dehydratase	2.30	1.2		All	*fabA*→BTH_I2042
BTH_I2039 (*lpxA*)	acyl- UDP-N-acetylglucosamine O-acyltransferase	3.73	1.9		All	*fabA*→BTH_I2042
BTH_I2190 (*wcaG*)	nucleoside-diphosphate-sugar epimerase	2.64	1.4		All	
BTH_I3150 (*lptE*)	lipoprotein	3.48	1.8		All	[33]
**Isoprenoids and siderophores biosynthesis (5)**						
BTH_I0783 (*ispH-1*)	4-hydroxy-3-methylbut-2-enyl diphosphate reductase	2.30	1.2		Bp, Bm	
BTH_I2418	siderophore related peptide synthetase			200	All	
BTH_II1233	putative siderophore non ribosomal peptide synthase			100	Bp	
BTH_II1236	putative non ribosomal peptide synthase/polyketide synthase			175.4	Bp	
BTH_II2243 (*ispH-2*)	4-hydroxy-3-methylbut-2-enyl diphosphate reductase	2.83	1.5		All	
**Other outer membrane proteins (7)**						
BTH_I1212	rare lipoproptein B	2.64	1.4		All	
BTH_I1907	uncharacterized lipoprotein	2.46	1.3		All	
BTH_I2443	Multidrug efflux protein	2.46	1.3		All	BTH_I2443→I2445 [33]
BTH_I2444	multidrug efflux protein (AcrB)	2.30	1.2		All	BTH_I2443→I2445 [33]
BTH_I2445	membrane fusion protein (AcrA)	2.83	1.5		All	BTH_I2443→I2445 [33]
BTH_II1520	OmpC, outer membrane protein	5.66	2.5		All	
BTH_II1720	OmpC, outer membrane protein	2.30	1.2		Bp, Bc	

a FC = fold change in differential expression;

b log_2_(FC) = logarithm_2_ of fold change.

c DAve = Difference in Average (amount); calculated as in Experimental procedures.

d Conservation in *Burkholderia* species pathogenic for humans: Bc = *B. cenocepacia*; Bm = *B. mallei*; Bp = *B. pseudomallei.*

e in the reference indicated, the gene or its orthologous has been found with the same trend of expression in growth conditions comparable with this work. Genes and corresponding proteins found to be differentially expressed both in transcriptomics and proteomics experiments are underlined.

### Regulation of Gene/Protein Expression by Temperature

The most striking effect of growth at 37°C versus 28°C is the lower expression level of genes related to cellular motility and chemotaxis: altogether, 12 genes involved in flagellar motility and 12 genes encoding chemotactic receptors and signal transduction proteins were expressed at lower levels at 37°C. On the other hand, three flagellar genes (*fliN*, *fliH*, *flgI*) were upregulated at 37°C: however, the *fliN* and *fliH* genes encode components of a type 3 secretion systems (T3SS) involved not only in translocation of flagellar subunits, but also of the anti-σ^28^ factor FlgM. Thus, their increased expression can be understandable even in a context of flagellar downregulation, as part of the complex regulation typical of the flagellar apparatus [20]. Growth at 37°C also resulted in downregulation of 6 out of 9 genes in the Chromosome 2-located operon encoding one of the two ATP synthase complexes present in the *B. thailandensis* genome; of 3 genes belonging to an operon encoding nitrate reductase and of 3 genes encoding nitrous oxide reductase subunits. Finally, 6 genes whose products belong to the class of universal stress proteins also showed lower expression at 37°C. Very few genes were upregulated by growth at 37°C: in addition to the T3SS proteins involved in the flagellar system, only two chaperones (*dnaJ* and *dnaK*) and the extracellular enzyme acid phosphatase were found to be transcribed at higher levels at 37°C. Interestingly, conditions identical to the ones studied in this work (28°C vs. 37°C, stationary phase cells) were included in a recently published investigation of *B. pseudomallei* genome expression in response to a large variety of environmental signals [21], which, in agreement with our results, showed that expression of chaperone-encoding genes was higher at 37°C, while most flagellar genes were downregulated at this temperature.

To confirm the results of the global gene expression experiments, and to investigate expression changes of cell surface-associated proteins, which, being in direct contact with the external environment, can play an important role in adaptation to the human host, we performed an enrichment procedure for outer membrane and cell surface-associated proteins (see *Methods*). Protein production in outer membrane protein-enriched preparations (OMPs) from BtCDC272 cultures grown at either 28°C or 37°C was analyzed by Mass Spectrometry, using Multidimensional Protein Identification Technology [22]. MudPIT analysis allowed us to verify the effective enrichment of known outer membrane proteins in the OMP fractions in all samples tested; Outer Membrane Proteins found to be differentially expressed (DEPs) are listed in File S8 (37°C versus 28°C; 54 DEPs) and File S9 (37°C An versus 37°C; 127 DEPs). Among proteins showing differential production, MudPIT analysis detected lower amounts of flagellin (the main flagellar subunit) from BtCDC272 grown at 37°C, in agreement with decreased transcription levels of the *fliC* gene (Table 1). The amounts of FliF and FlgE, two proteins belonging respectively to the flagellar hook and to the MS (membrane and supramembrane) ring, were also decreased at 37°C (Table 1), further suggesting downregulation of flagellar production at this temperature. In OMPs from BtCDC272 grown at 37°C, we found higher amounts of two proteins (BTH_I2962 and BTH_I2963) belonging to a type 6 secretion system (T6SS) [23] and also the GroEL subunit of the GroEL-GroES chaperonin (Table 1). Enhanced GroEL protein production at 37°C, together with higher transcription levels of *dnaJ* and *dnaK* (Table 1), would suggest a general temperature-dependent increase of chaperone production, consistent with their role as heat-shock proteins.

### Regulation of Gene/Protein Expression by Oxygen Limitation

Growing BtCDC272 at 37°C in oxygen-limiting conditions had a much larger impact on BtCDC272 physiology, inhibiting growth rate (Figure S1) and resulting in a larger number of DEGs (Table 2). In anoxic conditions, expression of 5 genes involved in sulfur assimilation and hydrogen sulfide production was reduced, likely in response to changes in the redox state of the cytoplasm due to lower oxygen levels. Likewise, transcription of the six genes encoding universal stress proteins, which already showed reduced expression at 37°C compared to 28°C, were further downregulated. In contrast with our observations, genes encoding universal stress proteins have been found to be upregulated in hypoxia in different *Burkholderia* species as well as in *Pseudomonas aeruginosa* [24]–[26]. A possible explanation for this discrepancy is that we compared cells in stationary phase rather than exponentially growing cells growing at different rates: indeed, it has been suggested that universal stress proteins respond to reduction in growth rate [27]. In addition, regulation mechanisms of Usp-encoding genes can vary in different bacteria; for instance, gene array experiments showed anaerobic downregulation of the *uspA* gene in *E. coli* [28], consistent with its role in response to oxidative stress in this bacterium [27].

Genes whose products show homology to pilus assembly components and regulators were also expressed less in anoxic conditions. MudPIT protein content analysis of the OMP cell fraction confirmed reduced production of BTH_I0090 (a putative regulator of pilus assembly) and BTH_II1990 (a putative outer membrane protein), in agreement with reduced transcription levels of their corresponding genes. Finally, MudPIT analysis indicated reduced levels of flagellin, of another putative outer membrane protein (BTH_II1638) and of three polypeptides involved in polyhydroxyalkanoate (PHA) synthesis (Table 2), whose presence in the OMP fraction is possibly due to their association to high molecular weight PHA granules.

Consistent with the lack of a nitrate source able to support BtCDC272 anaerobic growth in LB medium (data not shown), we did not observe any induction of genes involved in nitrate reduction and anaerobic respiration in oxygen-limiting conditions. Oxygen limitation induced two distinct protein secretion systems: one operon (BTH_I2959-BTH_I2965, with 6 out of 7 genes found to be differentially expressed in RNA-seq experiments) encodes a T6SS, whose outer membrane-associated components have also been found in higher amounts in BtCDC272 grown at 37°C compared to 28°C (Table 1). A second operon (BTH_I3204-BTH_I3206), encodes a protein export system showing high similarity to a system found in *Yersinia pestis* and to the *E. coli csg* complex, responsible for secretion and assembly of thin aggregative fimbriae (curli) on the outer membrane [29]. Interestingly, a large number of the genes showing increased transcription levels in oxygen-limiting conditions encodes proteins central to the cell’s primary metabolism: all ribosomal proteins and RNA polymerase subunits, nucleotide biosynthesis enzymes, and several enzymes involved in the biosynthesis of lipopolysaccharide (LPS) and of EPS precursors. Consistent with upregulation of energy-requiring cellular processes, the ATP synthase-encoding operon located on Chromosome 1 was also induced in response to oxygen limitation (Table 2). Higher transcription of chaperone-encoding genes in oxygen limitation (5 genes, Table 2) would also correlate with increased protein synthesis rates. In contrast, no increased expression for genes involved in DNA replication and cell wall biosynthesis was detected, as expected for cells in stationary phase. Increased expression of genes involved in primary metabolism, although apparently surprising, might be related to different metabolic requirements in stationary phase cells grown in anoxic conditions, as further discussed in “*Validation of functional genomics experiments by Real-Time PCR*”.

Protein analysis of the OMP fraction, performed on a separate set of cultures, confirmed the trends highlighted by RNAseq experiments (Table 2). Among cell surface-associated proteins, MudPIT analysis suggested increased production, in oxygen-limiting conditions, of the products of the BTH_I2962, BTH_I2963 and of BTH_I3205 genes, *i.e.*, components of a T6SS and of the *csg*-like protein secretion systems, in agreement with RNA-seq data (Table 2). Three proteins belonging to the flagellar structure (FliF, FlgE and FliD) were also found in higher levels in OMPs from BtCDC272 grown in oxygen-limiting conditions, in contrast to the main flagellar subunit, FliC, which was present in lower amounts (Table 2), possibly suggesting that the flagellar structure might be reorganized in response to lack of oxygen. Many DEPs found in the OMP fraction in anoxic conditions are cytoplasmic, such as chaperones, ribosomal proteins, etc.: the presence of cytoplasmic proteins, especially when abundant and/or part of high molecular complexes likely to co-precipitate with cell membranes during fractionation, is normally observed in OMP fractions [30], [31]. In addition to products of genes showing higher transcription levels, three cytoplasmic proteins belonging to non ribosomal peptide synthesis (NRPS) complexes were found in larger amounts in OMP fractions of BtCDC272 grown in oxygen-limiting conditions. NRPS activity can be aimed to production of antibiotics, signal molecules, and siderophores: indeed, BTH_I2418 shows high similarity to genes involved in siderophore biosynthesis such as ornibactin in *B. cenocepacia*, enterobactin in *E. coli* and pyoverdin in *P. aeruginosa*, thus suggesting that a siderophore-producing NRPS might be activated in anoxic conditions in BtCDC272.

### Validation of Functional Genomics Experiments by Real-Time PCR

**Figure 2:**
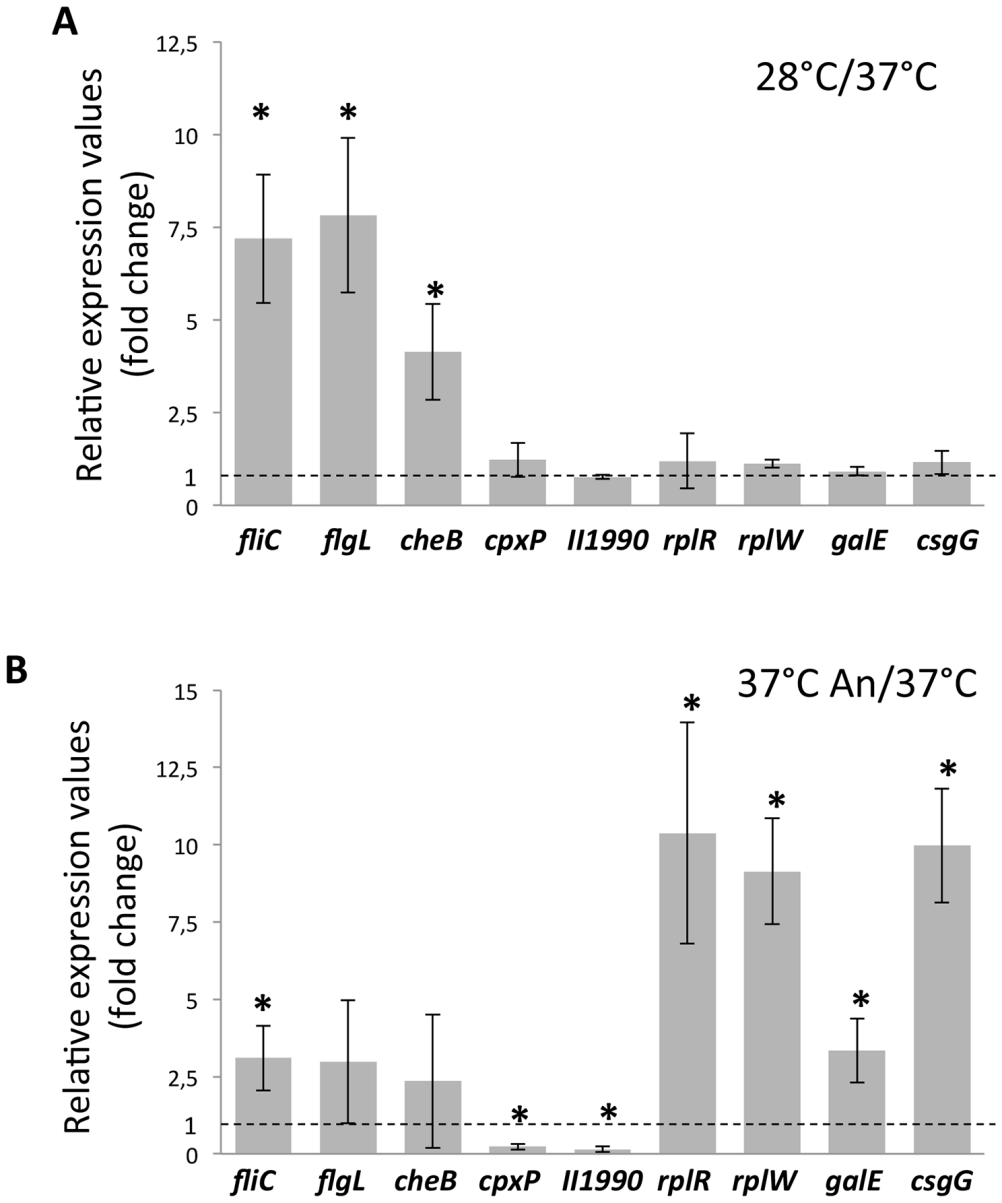
Validation of RNAseq results by qRT-PCR experiments. Genes tested are indicated in the graph; “II1990” refers to the BTH_II1990 gene. ΔCt between the gene of interest and the 16S gene was arbitrarily set at 1 (dashed line) for expression levels in BtCDC272 grown at 37°C in aerobic conditions, to which expression levels at either 28°C (Panel A) or at 37°C in anoxic conditions (Panel B) were compared. The Relative expression values indicated in the graph are the average of at least four experiments (two repeats, each performed on duplicate samples, from two independent RNA extractions), and standard deviations are shown. The asterisks denote significant differences relative to BtCDC272 grown at 37°C in aerobic conditions (*p*<0.05; Tukey multigroup analysis).

In order to confirm the results shown in the previous section, we performed quantitative Real Time PCR (qRT-PCR) determination of transcript levels on genes showing differential expression in RNAseq experiments. qRT-PCR were performed on RNA samples taken from two independent overnight cultures. We selected 9 genes, representative of the major trends in differential expression, as observed in RNA-seq experiments, namely: flagellar motility and chemotaxis-related genes (*fliC*, *flgK* and *cheB* genes), showing temperature-dependent regulation (Table 1); ribosomal operons, EPS-related genes and genes encoding protein secretion systems (*rplR*, *rplW*, *galE* and *csgG* genes), showing increased expression in oxygen-limiting conditions (Table 2). Finally, we tested two genes encoding cell surface-associated proteins (*cpxP* and BTH_II1990), whose downregulation in oxygen-limiting conditions was confirmed by reduced levels of their corresponding proteins in OMP (Table 2). For the evaluation of qRT-PCR results, amount of transcripts in BtCDC272 grown at 37°C in aerobic conditions were arbitrarily considered as “standard expression levels”, to which the effects of growth either at 28°C or in oxygen limitation were compared (Figure 2). qRT-PCR experiments clearly confirmed that oxygen limitation stimulates expression of the *rplR*, *rplW*, *galE* and *csgG* genes, while downregulating *cpxP* and BTH_II1990, in agreement with RNA-seq experiments (Figure 2B). As expected, none of this set of genes showed any significant expression change in response to different growth temperatures, suggesting that their expression specifically responds to anoxic conditions (Figure 2A). In contrast, expression of flagellar and chemotaxis-related genes showed a more complex pattern: *fliC*, *flgK* and *cheB* transcript levels were significantly higher at 28°C than at 37°C (Figure 2A), thus confirming the downregulation of these genes at 37°C observed in RNA-seq data (Table 1). However, a slight induction (2.35- to 3.10-fold) was also observed in anoxic conditions, although only the difference in *fliC* expression appears to be statistically significant (Figure 2B). This result is in apparent contrast with MudPIT observations that detected lower amounts of flagellin, the *fliC* gene product, in OMPs of BtCDC272 grown at 37°C An (Table 2): however, other flagellar genes show higher transcription levels in oxygen limiting conditions (Table 2), thus suggesting complex regulation of the flagellar machinery in response to anoxia.

**Figure 3:**
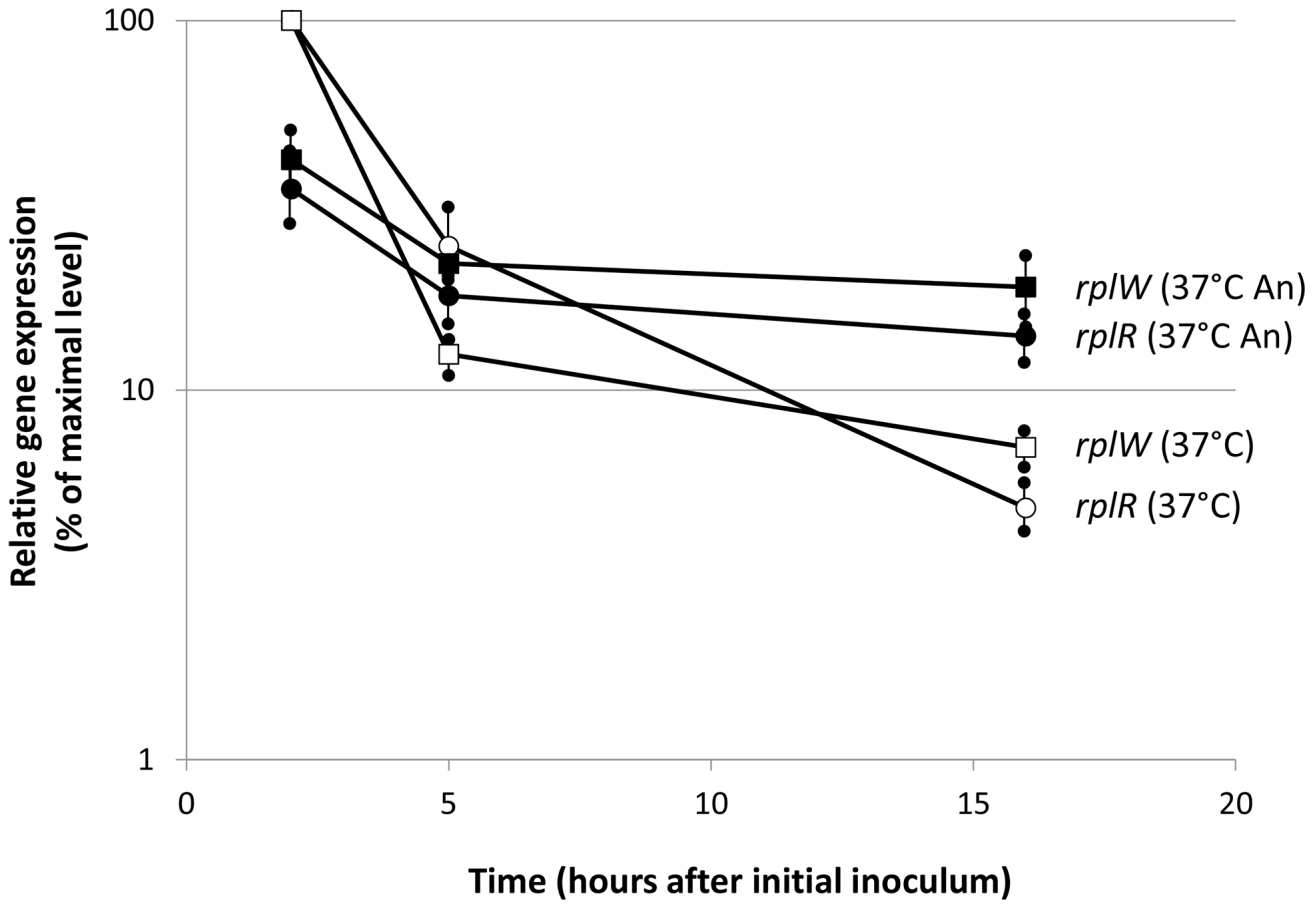
Time-course expression of ribosomal protein-encoding genes in different growth conditions. Samples were taken at 2, 5 and 16 hours from the initial inoculum at OD600 = 0.1 (see Figure S1A for comparison) and relative amounts of *rplR* (circles) and *rplW* (squares) transcripts were determined by qRT-PCR in cultures grown either aerobically (open symbols) and in oxygen limitation (closed symbols). Maximal gene expression corresponded to ΔCt values for *rplR* = 6.8 and for *rplW* = 5.5 and was set to 100%. qRT-PCR was repeated three times (with replicate samples for each experiment) on RNA obtained from a single extraction. Standard deviations are shown.

Results of qRT-PCR experiments confirmed increased expression of ribosomal protein-encoding genes in oxygen-limiting conditions in comparison to aerobic growth (Figure 2). Since genes encoding ribosomal proteins are known to be more expressed in optimal growth conditions, induction in anoxic conditions was unexpected; in addition, this result might seem at odds with previous findings showing decreased expression of protein synthesis-related genes in response to hypoxia in different *Burkholderia* species [25], [26]. In these reports, however, experiments had been carried on using exponentially-growing bacteria. To address these discrepancies, we performed qRT-PCR experiments measuring *rplR* and *rplW* expression at different stages of growth at 37°C either in aerobic or anoxic conditions (Figure 3). Cells were inoculated to OD_600nm_ = 0.1 and RNA samples were taken at 2, 5, and 16 hours. At the 2 hour sample, expression of both *rplR* and *rplW* was higher (ca. 4- and 3-fold, respectively) in the faster-growing aerobic cultures than in oxygen-limiting conditions. However, in aerobic cultures, a 10-fold decrease already took place at 5 hours, in correspondence to a shift to slower growth (see Figure S1A for comparison), followed by a further, albeit slighter, decrease at 16 hours (Figure 3). In contrast, although a reduction in *rplR* and *rplW* expression, as cells progressed into stationary phase, could also be observed in anoxic conditions, the decrease was not as steep, leading to residual *rplR* and *rplW* expression higher than in aerobic conditions, in agreement with RNAseq, MudPIT analysis and qRT-PCR experiments (Table 2, Figure 2). Thus, although maximal transcript levels for *rplR* and *rplW* were observed in exponential phase in aerobic conditions, as expected, their residual expression in stationary phase was higher under oxygen limitation, suggesting that a higher rate of protein and macromolecular synthesis might take place in these conditions.

### Temperature-dependent Regulation of Cell Motility

**Figure 4:**
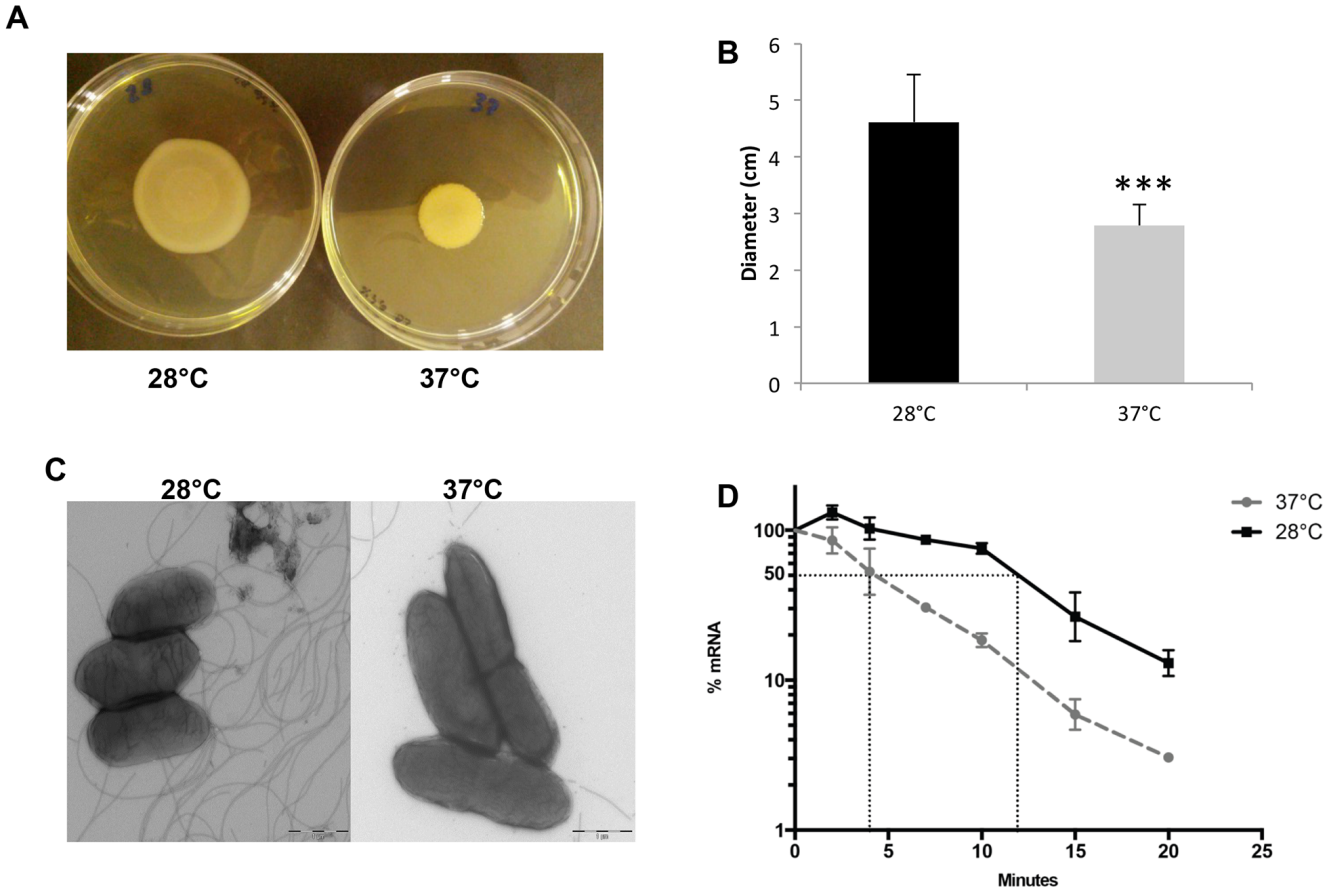
Temperature-dependence of cellular motility and *fliC* gene regulation. Motility assays on LB soft 0.4% agar plates as from a typical experiment (**A**) and quantitative estimate from an average of six independent experiments (**B**). Statistical analysis (two-tailed Student’s t test) of the results provided a p value<0.0001 (indicated by the three asterisks in the figure). **C**: TEM observation of cells from BtCDC272 overnight cultures grown either at 28°C or 37°C. The 1 μm scale bar is shown in the right bottom corner of each panel. **D**: RNA stability assays on the transcript of the *fliC* gene, as determined using qRT-PCR. The horizontal dashed line indicates 50% of residual amount of mRNA after rifampicin addition; the vertical dashed lines define the half-life values. Values are the average of four experiments (two repeats, each performed on duplicate samples, from two independent experiments) and standard deviations are shown.

Our results clearly indicate lower expression of flagellar and chemotaxis genes, and reduced production of flagellar proteins, in BtCDC272 grown at 37°C in comparison to 28°C (Table 1, Figure 2A), suggesting that flagellar motility would be subject to temperature-dependent regulation. To verify this hypothesis, we performed cell motility assays on soft agar plates, which, indeed, showed that BtCDC272 motility at 37°C was reduced by ca. 50% compared to 28°C (Figure 4A–B). Direct TEM observation provided further confirmation of a reduced number of flagella at 37°C (Figure 4C); however, in agreement with the results of MudPIT analysis (Table 1), TEM observations show that flagellar production is indeed downregulated, but not totally abolished, at 37°C. In order to better understand the contribution of flagella to cell motility in our assays, we tried to introduce mutations in the *fliC* gene of BtCDC272: however, despite various attempts, we did not succeed in creating any mutant (either in *fliC* or in other genes, data not shown), suggesting that the BtCDC272 is not readily amenable for genetic manipulations. Most flagellar genes in *B. thailandensis* are clustered in complex operons, present in two copies (one on each chromosome); temperature-dependent regulation seems to predominantly affect the flagellar operons located on Chromosome 1 (Table 1). The *fliC* gene (BTH_I3196), encoding flagellin, *i.e.*, the main flagellar subunit, is a direct target of temperature-dependent regulation (Table 1, Figure 2A). We investigated whether downregulation of *fliC* expression at 37°C might be mediated by reduced transcription initiation or by decreased mRNA stability. RNA degradation kinetics experiments performed by qRT-PCR showed a 3-fold decrease of *fliC* mRNA half-life at 37°C: estimated half-life was 4 minutes versus 12 minutes at 28°C (Figure 4D), suggesting that control of mRNA stability may account for, or at least contribute to, temperature-dependent regulation of flagellar genes.

### Polyhydroxyalkanoate (PHA) Accumulation does not Occur in Oxygen-limiting Conditions

**Figure 5:**
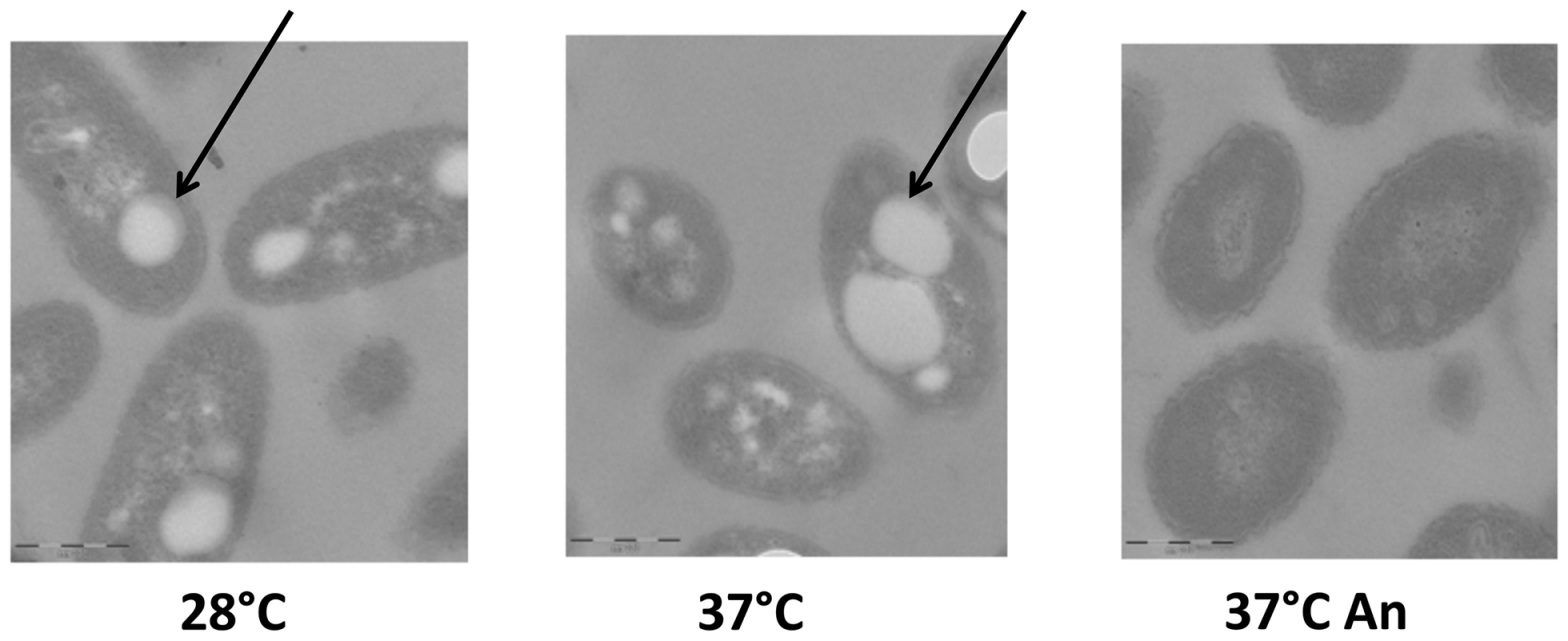
TEM observation of ultrathin sections of paraffin-embedded BtCDC272 grown in different conditions. Arrows point to PHA granules. The 1 μm scale bar is shown in the left bottom corner of each panel.

In response to nitrogen or phosphorous limitation, several bacterial species of the *Pseudomonas* and *Burkholderia* genera can store excess carbon as PHA. PHA accumulation results in the formation of large, light-reflecting granules easily detectable in the cytoplasm by electron microscopy (reviewed in [32]). Our proteomics data indicate that expression of proteins involved in PHA synthesis is downregulated in oxygen-limiting conditions (Table 2). However, previous reports had suggested increased transcription levels of genes involved in PHA biosynthesis in response to lack of oxygen in other *Burkholderia* species [25], [26], [33]. Thus, to confirm the results of our proteomic analysis, we assessed formation of PHA granules in BtCDC272 grown in different conditions by TEM observations. As shown in Figure 5, BtCDC272 cells grown in full aeration, regardless of temperature, showed significant accumulation of PHA granules, which were totally absent in cells grown in oxygen-limiting conditions. This result would be consistent with the notion that PHA synthesis is triggered by exhaustion of an essential nutrient in the presence of excess carbon. In the case of growth in oxygen-rich conditions, cells reach a much higher biomass in stationary phase (Figure S1), likely resulting in complete depletion of some essential nutrient (possibly phosphorous in a nitrogen-rich medium such as LB) that, in contrast, would still be present in cultures grown in oxygen-limiting conditions, thus preventing induction of PHA biosynthesis.

### Growth in Oxygen Limitation Stimulates EPS Production

**Figure 6:**
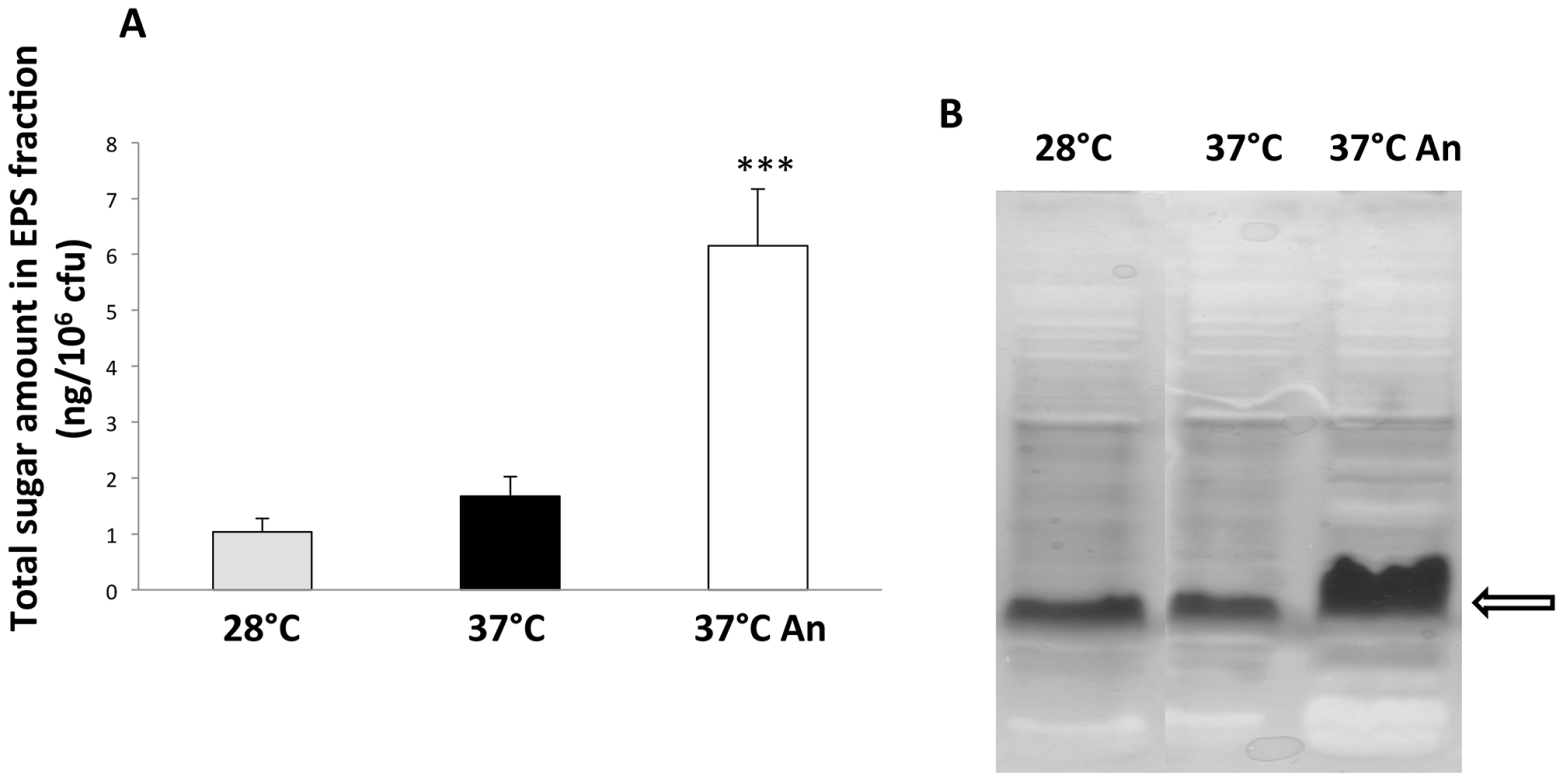
EPS/LPS determination. **A**) Quantitative determination of cell surface-associated polysaccharides using the phenol/sulfuric acid method. Values are the average of six measurements (two repeats for three independent cultures) and standard deviations are shown. Statistical analysis (two-tailed Student’s t test) of the results provided a p value<0.0001 (indicated by the three asterisks in the figure). **B**) PAGE analysis of cell surface-associated polysaccharides. The main band detected by silver staining, is indicated by the arrow. PAGE analysis of cell surface-associated polysaccharides from three independent BtCDC272 cultures gave very similar results.

RNA-seq experiments showed induction of several genes involved in LPS and/or EPS biosynthesis in response to oxygen limitation (Table 2). To confirm this result, we isolated cell surface-associated polysaccharides from BtCDC272 grown in different conditions by EDTA extraction, and quantified their amounts, using the phenol/sulfuric acid determination as described in *Methods* (Figure 6A). Amounts of EPS were roughly similar in BtCDC272 cultures grown either at 28°C or at 37°C in aerobic conditions (1.4 ng/10^6^ cfu versus 1.8 ng/10^6^ cfu, respectively), while, in contrast, they were significantly higher (5.6 ng/10^6^ cfu) at 37°C in oxygen-limiting conditions. We investigated whether the higher amount of cell surface-associated polysaccharides in BtCDC272 cultures grown in oxygen limitation could be ascribed to any specific cell structure. Indeed, SDS-tricine gels of cell-surface associated polysaccharides show a clear increase in production of LPS core (Figure 6B). Interestingly, increased LPS core production was only detected in the fraction obtained by EDTA treatment, while analysis of total cellular LPS did not show any significant difference in any growth condition tested (data not shown). Enhanced production of LPS core would be in agreement with RNA-seq data, which showed an anoxia-dependent increase in transcription levels for two LPS biosynthetic operons (BTH_I1470-1478 and BTH_I2035-2039, Table 2).

### *In vitro* Interaction of BtCDC272 Grown in Different Conditions with Innate Myeloid Cells

**Figure 7:**
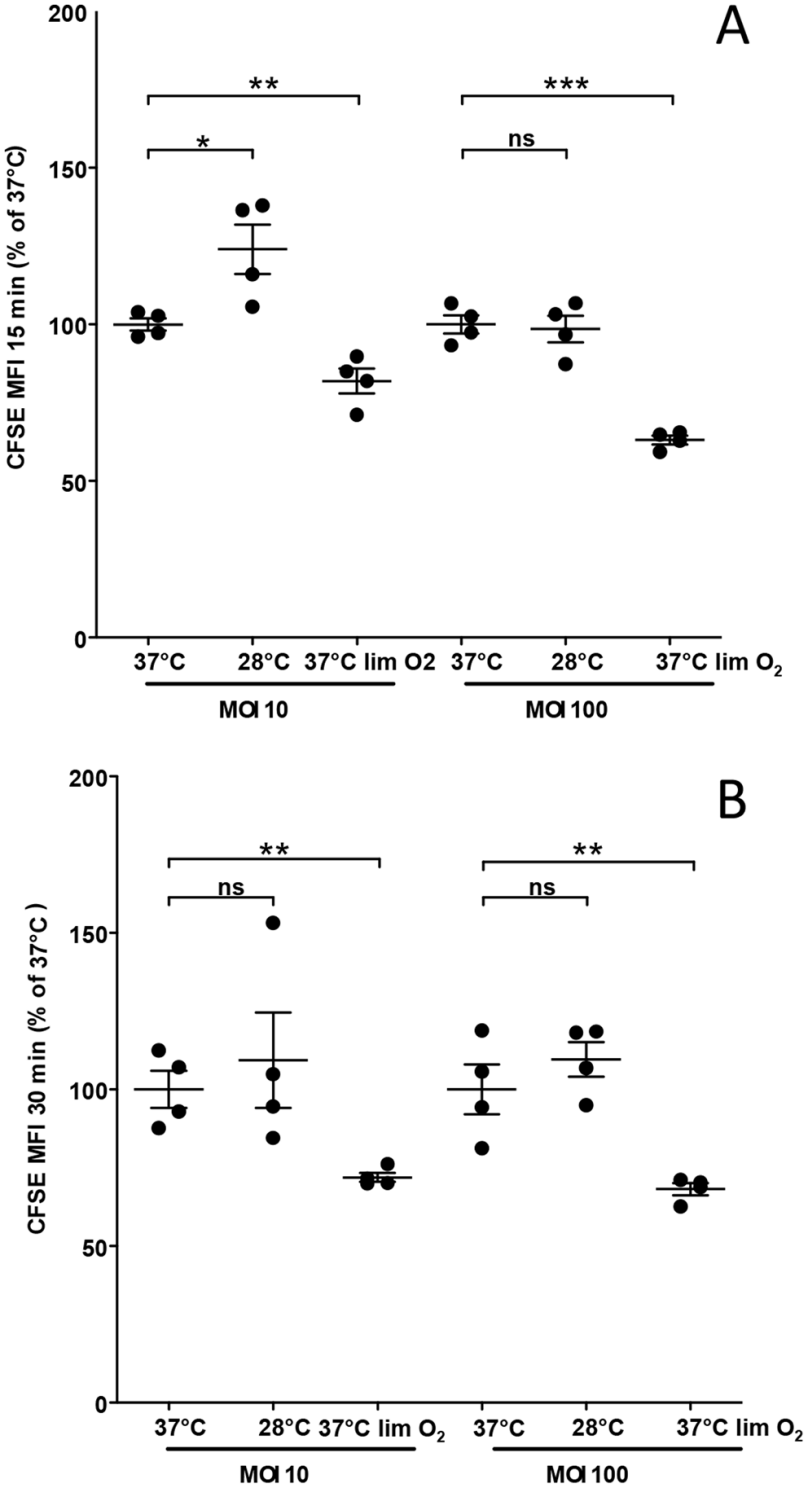
*In vitro* phagocytosis of BtCDC272 grown in different conditions by neutrophils in whole blood. *In vitro* phagocytosis of BtCDC (10 and 100 M.O.I) by human neutrophils of two donors was determined by FACS analysis at 15 min. Data are expressed as the percentage of control MFI. The mean value ± SEM is shown. *p≤0.05; **p≤0.01; ***p≤0.001; two-tailed Student’s *t* test. 0. Results for each donor, expressed as MFI, are shown in Figure S3.

Our results show that growth at 37°C and oxygen limitation, *i.e.*, environmental cues similar to those encountered upon contact to the human host, can affect a variety of cellular processes in BtCDC272. To investigate whether such changes in BtCDC272 cell physiology could impact its interaction with neutrophils, we measured *in vitro* uptake of BtCDC272 grown in different conditions by human neutrophils in whole blood (Figure 7). We monitored BtCDC272 uptake at two Multiplicities of Infection (10 and 100 M.O.I.) and at two different incubation times (15 and 30 minutes); BtCDC272 grown at 37°C in aerobic conditions was used as reference condition in these experiments. Similar levels of phagocytosis were obtained at 15 (Figure 7) and 30 minutes (data not shown), suggesting that bacterial uptake by neutrophils has already reached the plateau at 15 min. In comparison to cultures grown at 37°C, cells grown at 28°C, and thus characterized by increased flagellar production, were phagocytosed by human neutrophils with similar efficiency (MFI 34054±4325 *vs* 33789±4965 at 100 M.O.I, 15 min; Mean ± SEM) (Figure 7 and Figure S3). Only at 10 M.O.I. at 15 minutes did we detect a slight, although statistically significant, increase in BtCDC272 phagocytosis (24% increase, p = 0.02). In contrast, growing BtCDC272 in oxygen limitation significantly affected its phagocytosis by neutrophils at both incubation times and both M.O.I. tested (MFI 34054±4325 *vs* 21540±2837 at 100 M.O.I, 15 min; Mean ± SEM) (Figure 7 and Figure S3).

**Figure 8:**
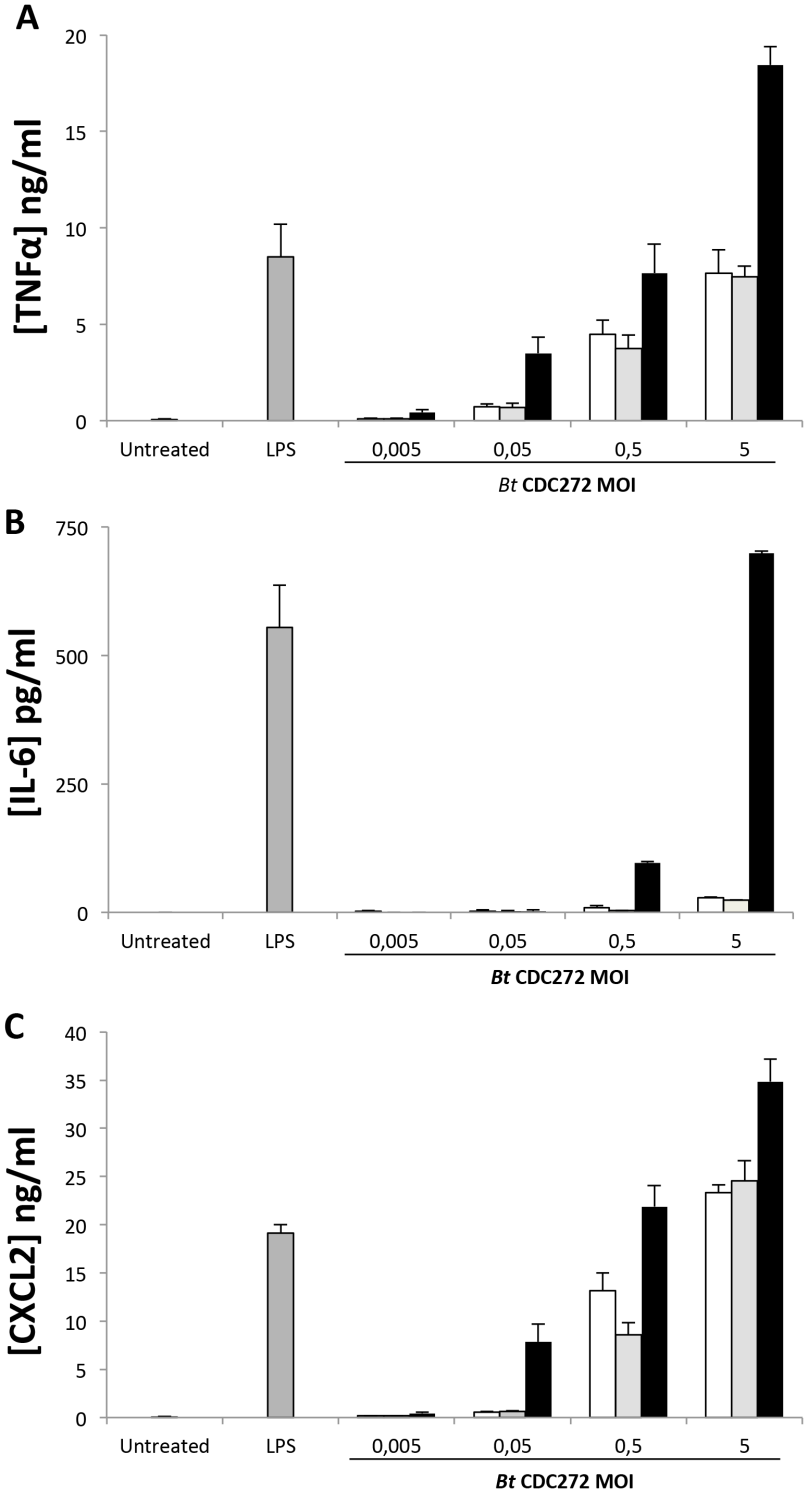
Cytokine production by mouse macrophage cell line RAW264.7 exposed to BtCDC272 grown in different conditions and at different M.O.I. Confluent monolayers of cells were incubated with bacteria cultured at the indicated conditions. Bacteria were added at MOI of 0.005, 0.05, 0.5 or 5 for 4 hours to confluent monolayers of RAW264.7 cells. LPS (100 ng/ml from *E. coli* Serotype 055:B5; Sigma-Aldrich) was used as positive control of macrophage activation. Murine CXCL2, TNF-α and IL-6 levels were measured in cell supernatants by ELISA Results are mean± SEM of two independent experiments.

To further evaluate possible effects of BtCDC272 on the inflammatory response, we monitored cytokine production in the culture supernatant of the mouse macrophage cell line RAW264.7 exposed to BtCDC272 grown in different conditions. Commercial preparation of *E. coli* LPS was used as positive control in macrophage activation experiments. As shown in Figure 8, exposure of macrophages to BtCDC272 grown in oxygen-limiting conditions resulted in a stronger secretion of tumor necrosis factor (TNF)-α, interleukin 6 (IL-6), and chemokine (C-X-C motif) ligand 2 (CXCL2), also known as macrophage inflammatory protein (MIP)-2. In particular, exposure to BtCDC272 grown in anoxic conditions triggered a dramatic increase in IL-6 production (Figure 8B). Macrophage viability was not significantly affected by exposure to BtCDC272, regardless of pre-growth conditions (data not shown), indicating that differences in cytokine production were indeed due to different levels of macrophage activation.

## Discussion

In this work, we have studied the *B. thailandensis* strain BtCDC272, a clinical isolate identified as the etiologic agent of a pleural infection in a 76-year old male [6]. Since *B. thailandensis*, unlike its closely related species *B. pseudomallei*, is normally non-pathogenic for humans, the BtCDC272 strain represents an intriguing example of a “transition state” between an environmental bacterium and a human pathogen. Analysis of its genome sequence did not provide any clear hint to its pathogenic potential: results of the genomic analysis confirmed that BtCDC272 produces an EPS more typical of strictly environmental *B. thailandensis* strains, rather than the Bp-like capsule polysaccharide produced by other *B. thailandensis* clinical isolates [10], thus suggesting that, in *B. thailandensis,* capsule production is not essential for pathogenesis in humans. Interestingly, several BtCDC272 genomic elements not present in the environmental isolate BtE264 were conserved in another clinical isolate, CDC3015869, including a putative prophage genome; although none of the 19 prophage-like genes show any clear homology to known virulence genes, it could be interesting to further evaluate the possible role of prophage-like sequences, which often carry pathogenesis-related genes, in *B. thailandensis* virulence. We then focused our investigation on the effects of different growth temperatures and oxygen availability, environmental signals associated to colonization of the human host, on gene expression and production of cell surface-associated proteins in BtCDC272. Our results suggest that temperature sensing plays a major role in flagellar production and cellular motility (Table 1, Figure 4), through a mechanism involving regulation of *fliC* gene expression at the mRNA stability level (Figure 4D). Downregulation of flagellar expression at 37°C has been observed in human pathogens, like in *Listeria monocytogenes* [34], and it is considered a strategy to prevent recognition of the highly antigenic flagellar structure by the host immune system. In contrast, mutations affecting flagellar motility reduce virulence both in *B. cepacia* [35] and in *B. pseudomallei* [36]. In *B. pseudomallei*, flagellar motility seems to be important for virulence when mice are infected via intranasal, but not intraperitoneal, route [36], [37]. Interestingly, however, the only obligate pathogen in the *Burkholderia* genus, *B. mallei*, is non-motile, likely as a result of pathoadaptive mutations inactivating its flagellar genes [13]. Thus, pathogenic *Burkholderia* species appear to have adopted very different strategies in terms of utilization of flagellar motility during host infection. Temperature-dependent downregulation of flagellar genes is also present in *B. pseudomallei,* as determined by recent gene expression studies [21], although with little or no effect on cell motility ([38] and our unpublished data). It could be speculated that modulation of flagella production at 37°C might somehow reduce the load of antigenic presentation to the host, without complete loss of cell motility, needed for host infection. In the BtCDC272 strain studied in our work, cellular motility appears to be the main cellular process affected by growth at 37°C versus growth at 28°C; indeed, apart from flagellar and chemotaxis genes, most of the genes showing reduced expression at 37°C belong to energy generation systems (*e.g.*, ATP synthase, nitrate and nitrous oxide reduction; Table 1), which might reflect an increased ATP requirement by the cell at 28°C due to higher flagellar activity. Interestingly, of the two operons encoding two distinct ATP synthase complexes present in the *B. thailandensis* genome, only the one located on Chromosome 2 was co-regulated with flagellar production and activity (Table 1). In contrast, the ATP synthase operon located on Chromosome 1 showed co-regulation with genes involved in primary metabolism (Table 2), thus suggesting possible functional specialization of the two ATP synthase complexes.

Growth in oxygen-limiting conditions showed a broader impact on BtCDC272 cell physiology, in terms of overall number of differentially expressed genes as well as of cellular processes affected (Table 2). Both RNA-seq and proteome analysis showed that protein secretion systems, an extracellular metalloprotease, and siderophore biosynthetic enzymes, are activated in response to anoxia (Table 2), suggesting that lack of oxygen might represent an important environmental signal for cellular processes linked to virulence. Interestingly, among the six T6SS present in the *B. thailandensis* genome, we found that only the one encoded by genes BTH_I2959-BTH_I2965 was activated by anoxia (Table 2). However, extracellular protein components of this T6SS complex (BTH_I2962-2963) were also found in higher amounts in the OMPs of BtCDC272 grown at 37°C compared to 28°C, although no difference in transcription level of the corresponding genes was observed (Table 1), suggesting that this T6SS might be subject to various forms of regulation in response to either temperature or oxygen limitation. This is in line with several observations suggesting that expression of T6SS-encoding genes in the *Burkholderia* genus is strongly dependent on environmental cues: for instance, a recent work has shown that expression of the cluster 1 T6SS in *B. mallei* (BMAA0730-0744) is activated by the two component regulatory system VirAG in response to lack of divalent metals such as iron and zinc [39]; in contrast, another study on *B. pseudomallei* reports that genes encoding two distinct T6SS are not transcribed in any of the 66 different *in vitro* conditions tested, suggesting that they are only expressed *in vivo*, possibly during macrophage infection [21].

Another important effect of oxygen limitation was the increase in expression of genes and proteins involved in primary metabolism, such as transcription and translation, ATP synthesis (operon located on Chromosome 1), and nucleotide biosynthesis (Table 2). However, anoxia-dependent increase in transcription of primary metabolism genes, such as the ribosomal protein-encoding genes *rplR* and *rplW*, is restricted to their residual expression levels observed in stationary phase, as their maximal expression occurs, consistent with their functional role, during exponential growth in aerobic conditions (Figure 4). In our work, we compared cells that have entered stationary phase due to exhaustion of different elements, *i.e.*, oxygen in case of anoxic conditions (Figure S1B) and another nutrient, possibly phosphorous, in aerobic cells. This results in strong differences in stationary phase metabolism, such as PHA accumulation, exclusive of aerobically-grown cells (Figure 5), or higher residual levels of protein synthesis and primary metabolism, observed in oxygen limitation (Table 2, Figures 2–3).

Anoxic conditions strongly increased expression of genes involved in LPS/EPS production and in the biosynthesis of their precursors (Table 2). High expression level for LPS-related genes in response to oxygen limitation was confirmed by direct observation, on SDS-PAGE, of increased LPS core production (Figure 6). Interestingly, increase of LPS core production in response to anoxia was only detected in the cell surface fraction released upon mild EDTA treatment (Figure 6B): this would suggest that excess LPS core is loosely attached to the cell membrane, possibly due to active shedding from the bacterium. It is noteworthy that, in *B. cepacia*, genes involved in EPS biosynthesis are also induced in response to oxygen limitation [33]; thus, induction of cell surface-associated polysaccharides in response to anoxia might be a conserved mechanism in different *Burkholderia* species. It would be tempting to speculate that increase in LPS production in BtCDC272 grown in oxygen limiting conditions (Figure 6B) might be the main trigger for the strong production of inflammatory mediators observed in macrophages (Figure 8). In addition to cytokine induction, neutrophil phagocytosis of BtCDC272 grown in oxygen limitation was also dampened (Figure 7), suggesting that growth in anaerobic conditions might increase virulence of BtCDC272. Our results about interaction between BtCDC272 and host innate cellular immunity also reiterate that, to fully evaluate interaction of bacteria with eukaryotic cells *in vitro*, it might be advisable to pre-grow them in conditions reflecting the host-bacterial interaction. We propose that oxygen limitation might take place upon ingestion of *B. thailandensis* by nematodes and other invertebrates, and it might therefore have evolved into an important environmental cue for active production of core LPS, which, through its endotoxin activity, might significantly contribute to virulence in these hosts.

## Methods

### Bacterial Growth Conditions and Motility Assays

For global gene and protein expression profiles analysis, BtCDC272 was grown in Luria Bertani (LB) broth in flasks filled for 1/5 of their volume and shaken at a rotation speed of 150 rpm, either at 28°C or 37°C. Oxygen-limiting conditions were obtained by growing BtCDC272 in the same conditions but with no shaking. The dissolved oxygen (DO) concentration was evaluated with a CellOx 325 membrane covered galvanic DO cell (WTW Scientific). The sensor was calibrated at the experimental temperature, which was controlled and monitored throughout the measurements. The DO values for LB medium prior to bacterial inoculums are in the range of the maximum DO for water (6.71 mg/l at 37°C).

Separate sets of cultures were used for each experiment performed in this study (RNAseq, proteomic analysis, qRT-PCR, electron microscopy observations, flagellar motility assays, EPS determination, interaction with blood cells). For each experiment, both “biological” (*i.e.*, different bacterial cultures) and “technical” replicates (*i.e.*, multiple measurements performed on the same biological sample) were obtained (see description of specific experiments for further details).

For motility test assays, 5 μl of BtCDC272 overnight cultures were inoculated on LB medium supplemented with 0.4% agar and incubated either at 28°C or 37°C for 16 hours. TEM analysis was performed as follows: 10 μl of an overnight culture of BtCDC272 grown either at 28°C or 37°C was absorbed on 300 mesh formvar/carbon copper grids; after 20 minutes the excess sample was dried with a filter paper and grids were negatively stained with 2% uranyl acetate. Sample observation was carried out at EFTEM Leo912 ab (Zeiss) transmission electron microscope operating at 80 kV and digital images were recorded by a Proscan 1 K CCD camera (Olympus).

For TEM observation of Epon resin-embedded samples, overnight cultures were centrifuged 30 minutes at 6000 *g*, resuspended in 2.5% gluteraldehyde in 0.1 M sodium cacodylate, incubated at ambient temperature for 15 minutes, centrifuged at 20000 *g* for 10 minutes. After an overnight incubation at 4°C, samples were treated with 1% OsO_4_ in distilled water for 30 min on ice in darkness. Finally, cells were dehydrated in a graded ethanol series (70, 80, 90, 96, and 100% ethanol), gradually infiltrated with Epon resin, sectioned (80-nm sections) using a Reichert Ultracut E Microtome (Leica Microsystems, Vienna, Austria), and collected on carbon-Formvar-coated 100-mesh hexagonal copper grids.

### Nucleic Acid Extractions

Genomic DNA from 1 ml of bacterial cultures was extracted with the GeneElute Bacterial Genomic DNA Kit (SIGMA), quantified using NanoDrop spectrophotometer (Nanodrop Technologies), and its integrity was evaluated by agarose gel electrophoresis. For RNA-seq experiments, total RNA was extracted and treated as previously described [40]. Total RNA extraction for qRT-PCR was performed using RNeasy minikit (QIAGEN) from 1 ml of BtCDC272 overnight cultures (ca. 16 hours growth, Figure S1). RNA samples were checked by agarose gel electrophoresis to assess the lack of degradation and then quantified with NanoDrop spectrophotometer. Genomic DNA degradation and reverse transcription were performed on 1 μg of total RNA using QuantiTect Reverse Transcription kit (QIAGEN).

### Genomic DNA Sequencing and Data Analysis

The library for genomic DNA sequencing was prepared according to the TruSeq DNA Sample preparation protocol (Illumina). Briefly, 1 μg of genomic DNA was sonicated to fragments with a medium length of 400 bp; after end repair, indexed adapters were ligated at DNA fragment ends, libraries were quantified using a quantification Real Time PCR (qPCR) by KAPA Library Quant Kits (KAPA Biosystems). After a short amplification step the library was sequenced on a Illumina Genome Analyzer IIx sequencer to generate 86 bp paired-end reads.

Paired-end reads were aligned against BtE264 genome (Ref Seq accession number: NC_007651 and NC_007650) using the aligner tool BWA [41] allowing up to 5 mismatches. High-quality alignment files (HQ-BAM) were generated by removing duplicates reads and by filtering-out reads with mapping quality less than 15 with samtools v0.1.19 [42]. HQ-BAM statistics on genome coverage and read depth were calculated using BEDTools [43] using annotation files provided by Burkholderia Genome database [44]. Single nucleotide variations (SNVs) were called using samtools v0.1.19 [42] filtering out those variations with a depth less than 20 reads and variant annotation were carried out using annovar [45]. *De novo* assembly of short reads was performed with AbySS [46]. In brief, the reads were assembled with different k-mers (from 41 to 64) and the assembly with k = 45 were chosen as optimal assembly for our dataset. Afterward, the contigs were annotated using FgenesB (Softberry) with default parameters and all the CDS deriving from automatic annotation were compared with BtE264 CDSs using BLAST suite.

The genomic data are available in Sequence Reads Archive (SRA) accession number SRR988099.

### RNA Sequencing and Data Analysis

RNA was extracted from two separate sets of BtCDC272 overnight cultures grown at 28°C, 37°C and 37°C anoxic. RNA samples with RNA Integrity Number (RIN) higher than 8 were employed for the RNA-Seq experiment. mRNA was enriched by removing ribosomal RNA using MICROBExpress kit (Ambion, Austin, TX); 500 ng were used for the preparation of cDNA libraries using the Ovation Prokaryotic RNA-seq System, which uses random primers selectively designed to avoid rRNA amplification. Then, 200 ng of double stranded cDNA were used to prepare NGS library with NuGEN’s Encore NGS Library System, according to the manufacturer’s protocol. Due to the small size of cDNA obtained, no fragmentation treatment was performed prior to library preparation, as suggested by NuGEN’s instructions. Each sample was prepared as a replicate and sequenced. Sequencing was performed using the Illumina Genome Analyzer IIx platform to generate paired-end 48 bp reads [40] (File S5).

Transcriptome reads were extracted using GERALD software and mapped on BtE264 genome by BWA applying the same parameters used for the genomic alignment. Read count for gene relative abundance, differential expression analysis and statistical analysis were performed as described [40]. Differential expression analysis was performed on a subset of 4658 CDSs (representing more than 80% of all the BtE264 CDSs) commonly above the Detection Threshold (7 reads) in all the growing conditions analyzed (File S5). The transcriptomic data are available in Sequence Reads Archive (SRA) accession number SRR988099.

### Multidimensional Protein Identification Technology (MudPIT) Analysis

The enrichment procedure for outer membrane proteins was basically carried out using the N-lauroylsarcosinate method as described [47]. Proteins were extracted from two separate cultures for each growth conditions, and analyzed separately. Outer membrane samples were first treated with RapiGest SF (Waters Corporation) at a final concentration of 0.2% (w/v). After incubation at 100°C for 5 min, the samples were cooled to room temperature and digested with trypsin (Sequencing Grade Modified Trypsin, Promega) as described previously [48]. Trypsin-digested samples were divided in half and analyzed as technical replicates by two-dimensional micro-liquid chromatography coupled to ion trap mass spectrometry (2DC-MS/MS) using ProteomeX-2 configuration (Thermo Electron Corporation, San Josè, CA, USA), according to [49]. In particular, peptides were eluted using eight steps of increasing ammonium chloride concentration (0, 20, 40, 80, 120, 200, 400, 700 mM).

### Mass Spectrometry Data Handling for MudPIT Analysis

The experimental mass spectra produced by MudPIT analyses were correlated to tryptic peptide sequences by comparing with theoretical mass spectra, obtained by *in silico* digestion of BtCDC272 protein database. Data processing was performed using the 3.3.1. Bioworks version, based on SEQUEST algorithm (Thermo Finnigan Corp.), and parameters previously reported [50].

The SEQUEST output were treated with an in-house software called MAProMa (Multidimensional Algorithm Protein Map) [51]: specific tools inserted in MAProMa permit the alignment of protein lists of replicate analyses, the evaluation of identification frequency and the subsequent comparison among the different conditions analyzed. Differentially expressed proteins are estimated by means of two algorithms of MAProMa, DAve (Differential Average), measuring changes in expression levels, and DCI (Differential Coefficient Index), measuring total protein amount in a given sample [22]. A DAve value either >0.1 or <−0.1 is an indication of different relative expression level between two samples, while a DAve value either = 200 or = −200 indicates exclusive presence of a protein in only one sample. Only proteins showing significant DAve values and identical trends in at least 3 out of 4 analyses (two technical replicates of samples from two independent cultures) were considered as differentially expressed.

### qRT-PCR and mRNA Decay Experiments

qRT-PCR was performed as previously described [47]. The primers used are listed in Table S1. All reactions were performed at least four times (duplicate repeats on RNA extractions from two independent cultures); RNA samples not treated with reverse transcriptase, used as negative controls, never showed significant threshold cycles. The relative amounts of the transcripts were determined using as reference genes 16S rRNA; for experiments shown in Figure 2, a different set of experiments using the the *aceA* mRNA as reference gene gave very similar results (data not shown). mRNA decay experiments on the *fliC* transcript were carried out using qRT-PCR as described [52]. Briefly, RNA was extracted from bacterial cultures in exponential phase (OD600 nm = 0.8); rifampicin at a 100 μg/ml final concentration was added at time 0, and additional samples (1 ml each from a 25 ml-culture grown in a 100 ml-flask) were taken at 2, 4, 7, 10, 15 and 20 minutes, and immediately processed as for standard qRT-PCR experiments. 16S rRNA was used as reference gene and, as expected, did not show any observable changes in threshold cycles following rifampicin treatment.

### Cell Surface-associated Polysaccharide Extraction and Extracellular Polysaccharide Analysis

Bacterial cultures grown in different conditions were normalized to OD600 = 2.5 and 100 ml of normalized cultures were centrifuged at 5000 rpm for 15 min at room temperature. Bacterial cells were resuspended in 2% EDTA (2 ml) and incubated for 4 h at 4°C under slight agitation. The suspension was centrifuged as above, and the supernatant was collected and filtered at 0.45 μm. Total sugar quantification was performed using the phenol-H_2_SO_4_ method [53]. After sugar quantification, samples were dried under vacuum and the lyophilized material was suspended in polyacrylamide gel electrophoresis (PAGE) sample buffer. LPS was separated by Tricine-sodium dodecyl sulfate (SDS)-PAGE and silver stained [54]. Both sugar quantification and PAGE analysis were performed on three separate cultures for each growth condition, always with identical results.

### *B. thailandensis* Phagocytosis Assay by Neutrophils in Fresh Human Blood

Phagocytosis assay of BtCDC272 by human neutrophils was performed as recently described [55]. Bacteria were pre-cultured in different growth conditions as described above and labeled with CFSE-mixed isomers (Invitrogen). 10^8^ bacteria were incubated for 1 h at room temperature with 5 μl CFSE 5 mM in 100 μl HBSS, extensively washed and resuspended in PBS with Ca^2+^/Mg^2+^. Labelling was controlled by FACS analysis. A total of 10^7^ (multiplicity of infection (MOI) of 10) or 10^8^ (MOI 100) CFSE-labelled BtCDC272 were added to 200 μl of whole blood (collected with heparin) and incubated for either 15 min or 30 min at 37°C in an orbital shaker. Human blood (3 ml/donor) was collected from 3 healthy blood donors after verbal consent. Written consent was not considered necessary by the institutional review board due to the lack of risk and invasiveness of the procedures involved. Participant consent was documented in the laboratory records. This study and the consent procedure were approved by the ethics committee of the Istituto Clinico Humanitas, Rozzano, Italy (14-11-2012). Samples were immediately placed on ice to block phagocytosis, and red blood cells were lysed by adding cold ammonium chloride lysis solution (3 ml, pH 7.2). Cells were stained with PerCP–anti-CD45 (BD Biosciences), fixed in 4% formaldehyde and CFSE mean fluorescence intensity (MFI) was analyzed in human neutrophils (CD45-positive cells, neutrophils defined as FSC-A^high^/SSC-A^high^) by FACS analysis [55].

### Murine Macrophages Activation and Cytokine Quantification by *B. thailandensis* Grown in Different Conditions

Mouse macrophage cell line RAW264.7 was cultured in RPMI 1640 medium supplemented with 10% Fetal Calf Serum and 2 mM L-glutamine (all from Invitrogen). Confluent monolayers of cells were incubated with bacteria cultured at the indicated conditions. Bacteria were added at MOI of 0.005, 0.05, 0.5 or 5 for 4 hours. LPS (100 ng/ml from *E. coli* Serotype 055:B5; Sigma-Aldrich) was used as positive control of macrophage activation. Murine CXCL2, TNF-α and IL-6 levels were measured in cell supernatants by ELISA (R&D DuoSet ELISA Development System) according to manufacturer’s instructions. Macrophage viability was determined as described previously [55].

## Supporting Information

Figure S1 **Growth curves and dissolved oxygen determination.** **A** Growth curves of BtCDC272 at 28°C (circles), 37°C (closed squares) and 37°C anoxic (open triangles). Data are from a typical experiment. Growth curves were repeated at least six times; standard deviations were less than 5%. **B**. Oxygen concentrations in liquid medium in cells grown at 37°C aerobically (squares) or in oxygen-limiting conditions (triangles). Dissolved oxygen values were determined as described in *Methods*.
(TIF)

Figure S2 **Functional enrichment of Differentially Expressed Genes (DEGs).** A) Functional categories enriched in 37°C vs 28°C DEGs list B) Functional categories enriched in 37°C An vs 37°C DEGs list. Dark blue columns represent BtCDC272 DEGs, and red columns indicate all BtE264 genes belonging to each functional category. (*P<0.05 or **P<0.01, binomial test; after Bonferroni correction).
(TIF)

Figure S3 ***In vitro* phagocytosis of BtCDC (10 and 100 M.O.I) by human neutrophils measured in each of the two donors.** Values are indicated in MFI. The average data, expressed as the percentage of control MFI, is shown in Figure 7.
(TIF)

Table S1 **Primers used for qRT-PCR experiments.** Coordinates refer to the BtE264 genome.
(DOCX)

File S1 **0X coverage region statistics and overlapping with Gi and nGi previously published.** i) sheet: “novel 0X coverage regions” contains the list of the regions with 0X coverage with respect to the BtE264 genome not including in the Gi-nGi dataset [10]. ii) sheet: “0X coverage regions in Gi-nGi” contains the list of the regions with 0X coverage with respect to the BtE264 genome that are included in the Gi-nGi dataset [10].
(XLS)

File S2 **List of the 929 CDS specific for BtCDC272 and relative annotation predicted by FGENESB.**
(XLS)

File S3 **List of the 331 BtCDC272-specific genes showing high similarity to CDS from a *Burkholderia* database containing 98044 protein sequences from 4 *B. mallei*, 9 *B. pseudomallei* and 3 *B. thailandensis* strains other than BtE264.**
(XLS)

File S4 **Non-synonymous SNVs distribution inside all the CDSs belonging to the Bt-EPS cluster and relative mutation rate evaluation.**
(XLS)

File S5 **RNA-seq mapping statistics, rRNA removal efficiency and analysis of gene detection sensitivity.**
(XLS)

File S6 **List of all the 416 Differentially Expressed Genes (DEGs) (p-val≤0.01 and log_2_ fold change ≥1 or log_2_ fold change ≤−1) in the of 37°C versus 28°C comparison.**
(XLS)

File S7 **List of all the 618 Differentially Expressed Genes (DEGs) (p-val≤0.01 and log_2_ fold change ≥1 or log_2_ fold change ≤−1) in the 37°C An versus 37°C comparison.**
(XLS)

File S8 **List of all the 54 Outer Membrane-associated proteins found to be differentially expressed in the 37°C versus 28°C comparison.** Significant differential expression was defined as described in *Methods* (Differential Average: DAve>0.2 and DAve<−0.2; Differential Coefficient Index: DCI>200 and DCI<−200).
(XLS)

File S9 **List of all the 127 Outer Membrane-associated proteins found to be differentially expressed in the 37°C Anoxic versus 37°C comparison.** Significant differential expression was defined as described in *Methods* (Differential Average: DAve>0.2 and DAve<−0.2; Differential Coefficient Index: DCI>200 and DCI<−200).
(XLS)
