# Supplementary figures and images for: Gene and Protein Expression in Response to Different Growth Temperatures and Oxygen Availability in Burkholderia thailandensis
Source: PLoS One. 2014 Mar 26;9(3):e93009. doi: 10.1371/journal.pone.0093009 (PMC3966863; doi:10.1371/journal.pone.0093009)

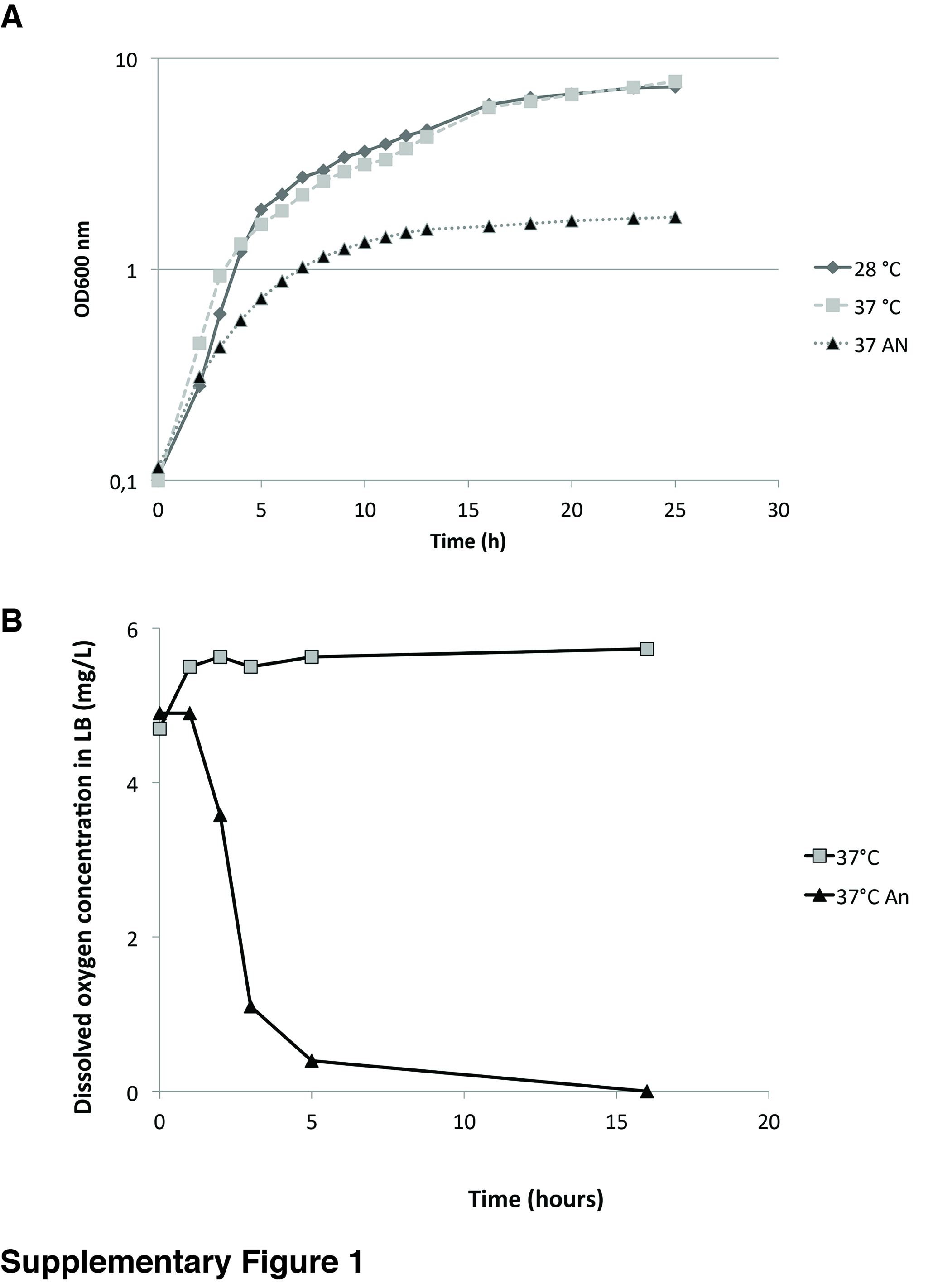

Supplement: Figure S1 — Growth curves and dissolved oxygen determination. A Growth curves of BtCDC272 at 28°C (circles), 37°C (closed squares) and 37°C anoxic (open triangles). Data are from a typical experiment. Growth curves were repeated at least six times; standard deviations were less than 5%. B. Oxygen concentrations in liquid medium in cells grown at 37°C aerobically (squares) or in oxygen-limiting conditions (triangles). Dissolved oxygen values were determined as described in Methods. (TIF) [file pone.0093009.s001.tif]

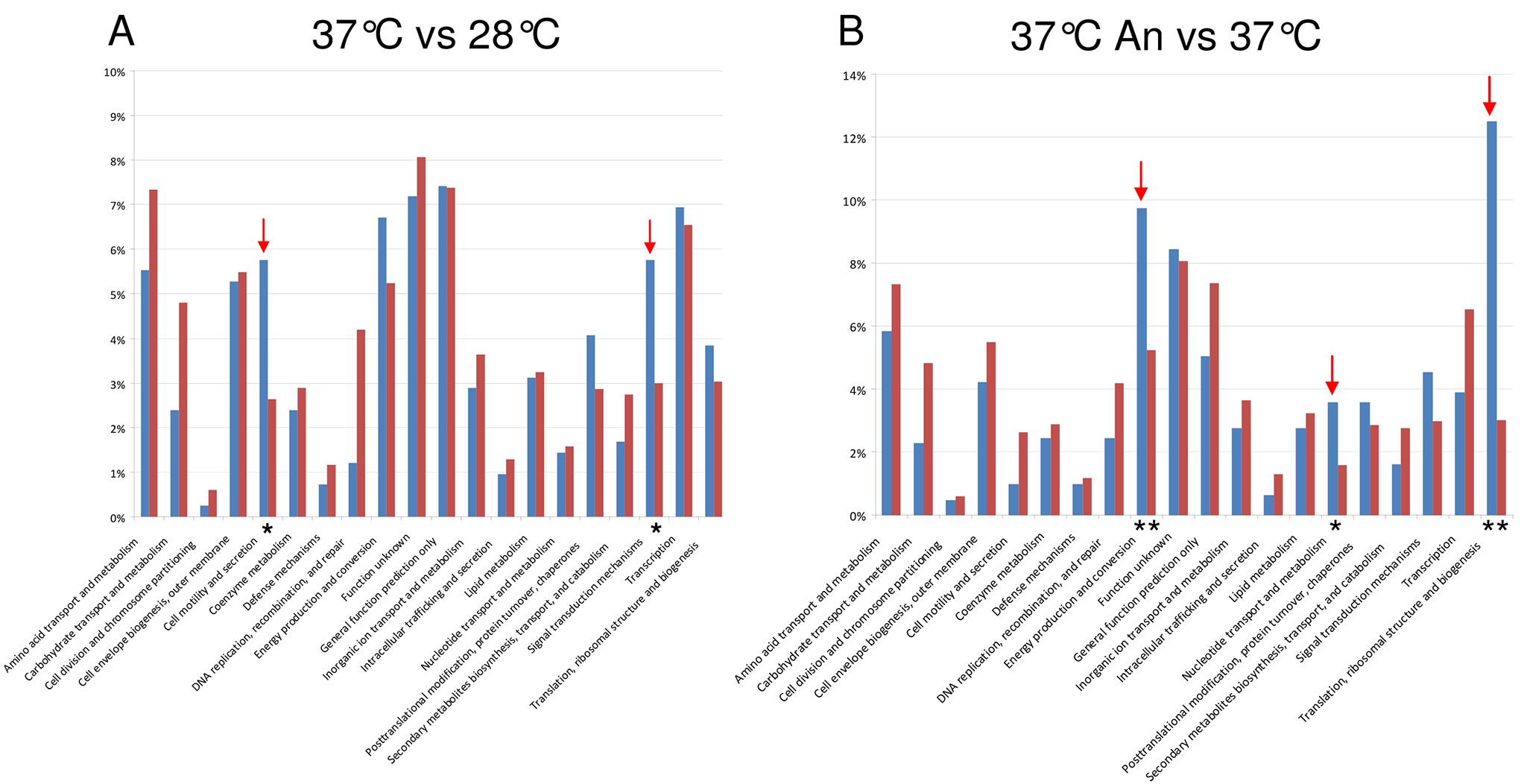

Supplement: Figure S2 — Functional enrichment of Differentially Expressed Genes (DEGs). A) Functional categories enriched in 37°C vs 28°C DEGs list B) Functional categories enriched in 37°C An vs 37°C DEGs list. Dark blue columns represent BtCDC272 DEGs, and red columns indicate all BtE264 genes belonging to each functional category. (*P<0.05 or **P<0.01, binomial test; after Bonferroni correction). (TIF) [file pone.0093009.s002.tif]

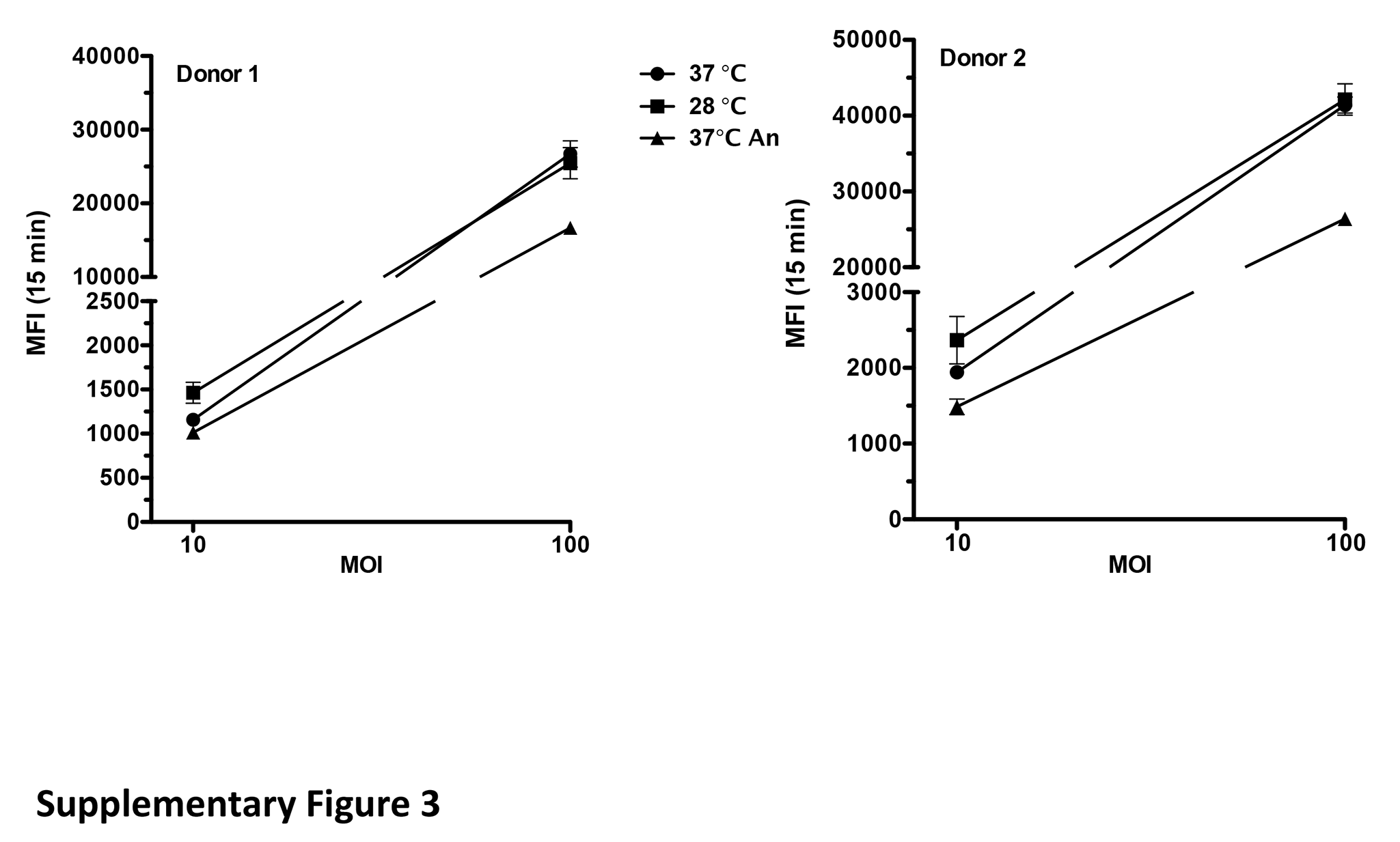

Supplement: Figure S3 — In vitro phagocytosis of BtCDC (10 and 100 M.O.I) by human neutrophils measured in each of the two donors. Values are indicated in MFI. The average data, expressed as the percentage of control MFI, is shown in Figure 7. (TIF) [file pone.0093009.s003.tif]
