# Supplementary material for: Gene and Protein Expression in Response to Different Growth Temperatures and Oxygen Availability in Burkholderia thailandensis
Source: PLoS One. 2014 Mar 26;9(3):e93009. doi: 10.1371/journal.pone.0093009 (PMC3966863; doi:10.1371/journal.pone.0093009)
Supplement: Table S1 — Primers used for qRT-PCR experiments. Coordinates refer to the BtE264 genome. (DOCX) [file pone.0093009.s004.docx]

**Supplementary Table 1**. Primers used for qRT-PCR experiments

| **Oligonucleotide** | **5′→3′ sequence** | **Coordinates^a^** |
| --- | --- | --- |
| 16S_F | TGTCGTCAGCTCGTGTCGTGA | 1238642-1238662 |
| 16S_R | ATCCCCACCTTCCTCCGGT | 1238765-1238747 |
| BT_aceA_RT_for | AGGAACTGCAAAAGCAATGG | 2262792-2262773 |
| BT_aceA_RT_rev | AGCGTGTGTTCTTGCTGGAT | 2262681-2262700 |
| BT_flgL_RT_for | GCAGTACACGCAGAACCAGA | 284987-285006 |
| BT_flgL_RT_rev | GCGTGCATGATCGACTGATA | 285097-285078 |
| BT_fliC_RT_for | TTCGCAGACGAACTACAACG | 3642825-3642844 |
| BT_fliC_RT_rev | GACATGCTTTGCGACAGGT | 3642941-3642923 |
| BT_cheB_RT_for | GAGCTGATCAAGCAGCACAA | 3624035-3624016 |
| BT_cheB_RT_rev | GTCAGCGACGAAACCATCAC | 3623905-3623924 |
| BT_rplR_RT_for | GAAGTTCGTGCGCAGCTC | 3493738-3493721 |
| BT_rplR_RT_rev | GAAGGCGACGGATTCGAT | 3493619-3493636 |
| BT_rplW_RT_for | CAAGAACGAGCAAGTCGTGT | 3500143-3500124 |
| BT_rplW_RT_rev | CGTTGACGGAATCAACTTCA | 3500040-3500059 |
| BT_csgG_RT_for | AAGTTCGACAACCGCTCGAGC | 3651791-3651771 |
| BT_csgG_RT_rev | CCTGCTTGATCTCGTCGAGGT | 3651638-3651658 |
| BT_galE_RT_for | CTTGGT TGACGGGAAGAAGA | 1671614-1671633 |
| BT_galE_RT_rev | CGGATGCAAGTTGTCCAT TA | 1671731-1671712 |

^a^Coordinates refer to *B. thailandensis* E264 genome (NCBI Accession Number NC_007651.1)
